# Progenitor potential of nkx6.1-expressing cells throughout zebrafish life and during beta cell regeneration

**DOI:** 10.1186/s12915-015-0179-4

**Published:** 2015-09-02

**Authors:** Aurélie P. Ghaye, David Bergemann, Estefania Tarifeño-Saldivia, Lydie C. Flasse, Virginie Von Berg, Bernard Peers, Marianne L. Voz, Isabelle Manfroid

**Affiliations:** Laboratory of Zebrafish Development and Disease Models (ZDDM), GIGA-Research, (Sart-Tilman) University of Liège, Avenue de l’Hôpital 1, B34, 4000 Liège, Belgium

**Keywords:** *nkx6.1*, *ascl1*, pancreas, duct, centroacinar cells, beta cells, stem cells, lineage tracing, multipotent progenitors, regeneration, diabetes, zebrafish, Notch, Wnt

## Abstract

**Background:**

In contrast to mammals, the zebrafish has the remarkable capacity to regenerate its pancreatic beta cells very efficiently. Understanding the mechanisms of regeneration in the zebrafish and the differences with mammals will be fundamental to discovering molecules able to stimulate the regeneration process in mammals. To identify the pancreatic cells able to give rise to new beta cells in the zebrafish, we generated new transgenic lines allowing the tracing of multipotent pancreatic progenitors and endocrine precursors.

**Results:**

Using novel bacterial artificial chromosome transgenic *nkx6.1* and *ascl1b* reporter lines, we established that *nkx6.1*-positive cells give rise to all the pancreatic cell types and *ascl1b*-positive cells give rise to all the endocrine cell types in the zebrafish embryo. These two genes are initially co-expressed in the pancreatic primordium and their domains segregate, not as a result of mutual repression, but through the opposite effects of Notch signaling, maintaining *nkx6.1* expression while repressing *ascl1b* in progenitors. In the adult zebrafish, *nkx6.1* expression persists exclusively in the ductal tree at the tip of which its expression coincides with Notch active signaling in centroacinar/terminal end duct cells. Tracing these cells reveals that they are able to differentiate into other ductal cells and into insulin-expressing cells in normal (non-diabetic) animals. This capacity of ductal cells to generate endocrine cells is supported by the detection of *ascl1b* in the *nkx6.1*:GFP ductal cell transcriptome. This transcriptome also reveals, besides actors of the Notch and Wnt pathways, several novel markers such as *id2a*. Finally, we show that beta cell ablation in the adult zebrafish triggers proliferation of ductal cells and their differentiation into insulin-expressing cells.

**Conclusions:**

We have shown that, in the zebrafish embryo, *nkx6.1+* cells are bona fide multipotent pancreatic progenitors, while *ascl1b+* cells represent committed endocrine precursors. In contrast to the mouse, pancreatic progenitor markers *nkx6.1* and *pdx1* continue to be expressed in adult ductal cells, a subset of which we show are still able to proliferate and undergo ductal and endocrine differentiation, providing robust evidence of the existence of pancreatic progenitor/stem cells in the adult zebrafish. Our findings support the hypothesis that *nkx6.1*+ pancreatic progenitors contribute to beta cell regeneration. Further characterization of these cells will open up new perspectives for anti-diabetic therapies.

**Electronic supplementary material:**

The online version of this article (doi:10.1186/s12915-015-0179-4) contains supplementary material, which is available to authorized users.

## Background

The pancreas is composed of an exocrine compartment with acinar and ductal cells, which secrete and transport digestive enzymes into the gut, and an endocrine compartment, which regulates glucose homeostasis by secreting pancreatic hormones into the bloodstream. Loss of pancreatic insulin-producing cells (beta cells) is a hallmark of diabetes. An attractive therapeutic approach to cure this disease is to stimulate beta cell regeneration in vivo from another pancreatic cell type or from progenitors. However, the regenerative capacity of beta cells is very limited in mammals and the cellular and molecular mechanisms involved need to be well understood before we will be able to stimulate this process. The zebrafish (*Danio rerio*), owing to its phenomenal capacity to restore beta cells after targeted cell ablation [1–3], has become an attractive model organism for the study of the regeneration process. To this end, tools need to be developed, especially to define the source of the new beta cells. So far, no zebrafish transgenic lines have been available to allow a lineage tracing of either multipotent pancreatic progenitors, giving rise to both exocrine and endocrine tissues, or pancreatic endocrine precursor cells.

The pancreas develops from two domains, called the dorsal bud and the ventral bud, which emerge from the foregut endoderm [4, 5]. In zebrafish, the dorsal bud generates the first wave of endocrine cells, which cluster at 24 hours post fertilization (hpf) to form the principal islet [6]. The ventral bud emerges anteriorly to the dorsal bud at 32 hpf and gives rise to acinar, ductal, and to a second wave of endocrine cells [4, 7, 8]. These late endocrine cells originate either from the extra-pancreatic ducts (EPDs) and contribute to the expansion of the principal islet [9–11] or from the intra-pancreatic ducts (IPDs) and form small secondary islets all along them. These IPDs contain pancreatic Notch-responsive cells (PNCs), which represent a population of progenitors of endocrine cells and ductal cells but not of acinar cells [7, 12].

Notch signaling pathway controls the differentiation of pancreatic cells both in zebrafish and mice (reviewed by [13]). One of its functions is to maintain a pool of progenitors in an undifferentiated state through the repression of genes of the achaete scute-like (ASCL) family or of the atonal-related protein (ARP) family. In the murine pancreas, Notch signaling prevents endocrine cell differentiation through the repression of *neurog3* [14]. In zebrafish, *neurog3* is not expressed in the pancreas and therefore the control of endocrine cell fate is fulfilled by other ASCL/ARP factors, namely Ascl1b and Neurod1, which are both repressed by Notch signaling [15]. Exactly like the inactivation of murine *Neurog3*, their simultaneous inactivation completely prevents endocrine cell differentiation leading to the loss of all hormone-secreting cells [15]. *ascl1b* is the earliest pancreatic marker identified during zebrafish development, its expression starting at the end of gastrulation in the prospective pancreatic region (10 hpf). *ascl1b* is transiently expressed during the formation of the dorsal bud (10–17 hpf) and, like murine *Neurog3*, is not detected in hormone-expressing cells. Later, in the ventral bud, *ascl1b* expression is turned on when the endocrine cell differentiation program is induced through the blocking of Notch signaling [7, 12, 16]. This Notch inactivation triggers a massive expression of *ascl1b* in IPDs [15]. These data suggest that *ascl1b* expression is restricted to the committed endocrine precursors. However, the observation that the onset of *ascl1b* expression in the prospective pancreatic region precedes all other known pancreatic progenitor markers raises the possibility of the multipotency of the first *ascl1b+* cells.

Another key factor for pancreatic development is the homeobox transcription factor Nkx6.1. In the mouse, it is expressed in the multipotent progenitors during early pancreatic development [17], and, in the zebrafish, *nkx6.1* is expressed early in the pancreatic primordium of the dorsal bud (from 11.5 hpf onwards) [18]. At later developmental stages in the mouse embryo, *Nkx6.1* becomes restricted to the endocrine/duct bipotential trunk domain [19]. Similarly, *nkx6.1* is first broadly expressed in the zebrafish pancreatic ventral bud primordium [20], then segregates from the *ptf1a*+ acinar cells to persist in the primitive ducts [20–22] that will give rise to the mature ducts and to secondary islets [7]. In the mouse, *Nkx6.1* is expressed in the differentiated beta cells [23] while in the zebrafish, *nkx6.1* is never expressed in beta cells nor in the other pancreatic hormone-expressing cells [18]. These data suggest that in zebrafish *nkx6.1* also marks multipotent pancreatic progenitors. However, previous findings suggested that the early ventral bud primordium was composed of a heterogeneous population of pancreatic cells comprising Notch-responsive cells, giving rise to ductal and endocrine cells, separated from the *ptf1a*+ cells, which generate the acinar cells [7]. This study raises the question of the identity of the multipotent pancreatic progenitors in the zebrafish ventral pancreatic bud and its derivatives.

Here, we show that *nkx6.1* labels multipotent pancreatic progenitors giving rise to all of the different pancreatic cell types (endocrine, ductal, and acinar) while *ascl1b* marks endocrine precursors leading to the different endocrine cell types. For this purpose, we have generated two novel bacterial artificial chromosome (BAC) transgenic *nkx6.1* and *ascl1b* reporter lines, *Tg(nkx6.1:eGFP)* and *Tg(ascl1b:eGFP-2A-creER*^*T2*^*)*, that both faithfully recapitulate the expression of the *nkx6.1* and *ascl1b* endogenous genes. Using these novel transgenic tools, we were able to analyze in detail the interdependency between these two factors and their relationship with the Notch signaling pathway. We also demonstrate that *nkx6.1* expression persists in the adult ductal tree, notably in the centroacinar/terminal end duct cells (CACs), for which we show that they are able to differentiate into insulin-expressing cells in vivo. By isolating *nkx6.1:*eGFP+ cells from the dissected pancreases of adult fish, we determined the transcriptome of adult pancreatic ductal cells, which revealed the expression of several regulatory genes potentially involved in endocrine regeneration. Finally, we provide evidence that regenerating beta cells also originate from ductal cells.

## Results

### The bacterial artificial chromosome reporter *Tg(nkx6.1:eGFP)* recapitulates in vivo the expression of the endogenous *nkx6.1* gene

**Fig. 1:**
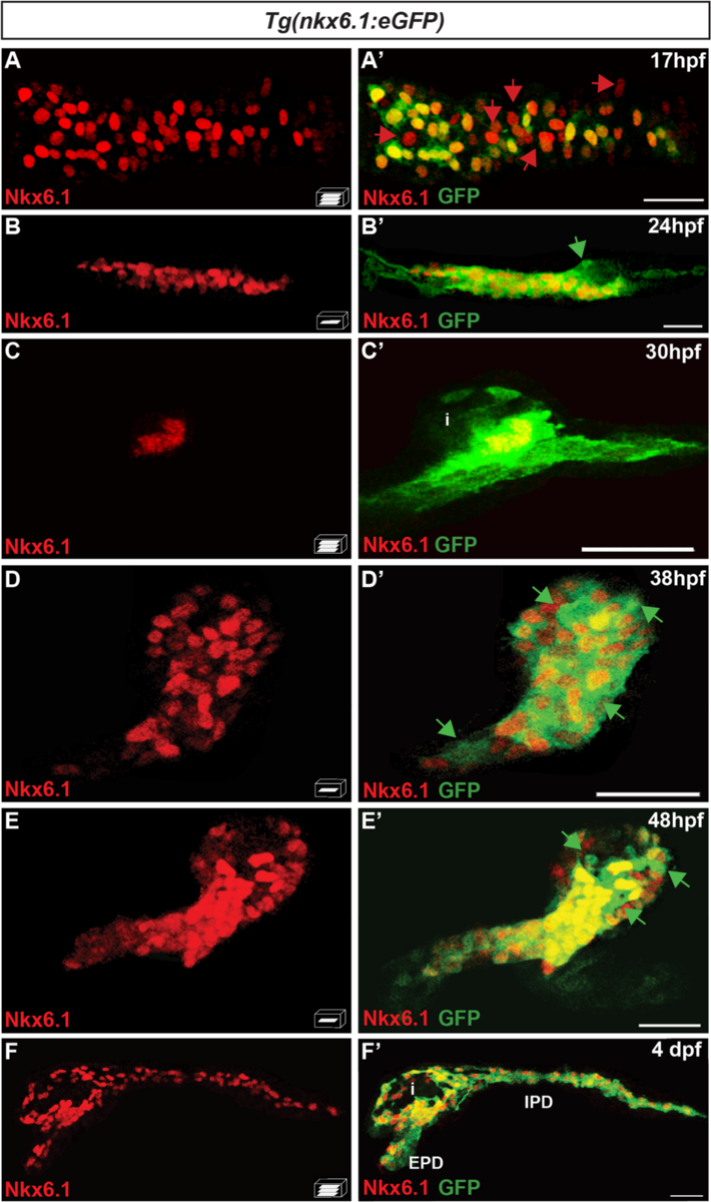
The bacterial artificial chromosome reporter line *Tg(nkx6.1:eGFP)* mirrors the expression of the endogenous *nkx6.1* gene. Immunodetection of endogenous Nkx6.1 (*red*) and GFP (*green*) in *Tg(nkx6.1:eGFP)* embryos of the indicated stages. *Green arrows* point to Nkx6.1–/GFP+ cells and *red arrows* to Nkx6.1+/GFP- cells. All views are either lateral (**b**, **b'**, **c**, and **c'**) or ventral (**a**, **a'**, **d**, **d'**, **f**, and **f'**) with the anterior part to the left. They represent either z-plane confocal images (**b**, **d**, **e**) or confocal projection images (**a**, **c**, **f**). Scale bars = 30 μm. *EPD* extra-pancreatic duct, *IPD* intra-pancreatic duct, *i* islet

To label the *nkx6.1*-expressing cells, we generated a transgenic line driving the expression of the enhanced green fluorescent protein (eGFP) under the control of *nkx6.1* regulatory regions. We engineered a BAC spanning from 55 kb upstream to 95 kb downstream of the *nkx6.1* gene and inserted the eGFP coding regions into exon 1, replacing the beginning of the *nkx6.1* open reading frame (Additional file 1: Fig. S1A). This BAC reporter construct was introduced into the zebrafish genome using the Tol2 transposon system [24, 25] and the stable transgenic line *Tg(nkx6.1:eGFP)* obtained showed expression of green fluorescent protein (GFP) in the nervous system and in the pancreas, which mirrors the endogenous Nkx6.1 protein expression (Additional file 1: Fig. S1B). Detailed comparison of the localization of these two proteins in the pancreas during development confirmed that GFP is indeed co-expressed with Nkx6.1 (Fig. 1). Indeed, together with the endogenous Nkx6.1 protein [18], GFP is expressed at the base of the endocrine islet at 24 and 30 hpf (Fig. 1b, c), in the ventral bud at 38 and 48 hpf (Fig. 1d, e), and in IPDs and EPDs at 4 days post fertilization (dpf) (Fig. 1f, f'). In contrast, at earlier stages, GFP was detected in a subset of Nkx6.1+ cells, probably due to the delay of GFP expression compared to Nkx6.1. Indeed, at 17 hpf, about 75 % of the Nkx6.1+ cells showed detectable GFP expression (Fig. 1a, a') and at 14 hpf, this proportion dropped even further to about 25–30 % (data not shown). Conversely, a few hours after the onset of *nkx6.1* gene expression, some GFP+/Nkx6.1– cells were also detected (*green arrows*, Fig. 1b'–e'). This GFP labeling is not the result of an ectopic expression of the *gfp* transcript, as double fluorescent whole-mount in situ hybridization (WISH) showed that the *gfp* transcripts are present in the same pancreatic domain as *nkx6.1* transcripts (data not shown) and importantly, like *nkx6.1*, *gfp* transcripts were not found in hormone-expressing cells (Additional file 1: Fig. S1C–C'', D–D''). Hence, prolonged GFP detection is rather due to the well-known high stability of GFP (±24 h half-life [26]), which persists in cells where Nkx6.1 protein is no longer found. This is nicely illustrated at 30 hpf, where strong GFP expression is detected at the base of the forming islet where Nkx6.1+ pancreatic progenitors are located, while weak GFP labeling is found dorsally within the islet, where differentiated endocrine cells, devoid of Nkx6.1, are clustered (Fig. 1c). As expected, this prolonged GFP detection will gradually fade away, finally to disappear completely in the differentiated endocrine cells (Fig. 1f).

The high stability of GFP allowed us to perform short-term lineage tracing to follow the immediate progeny arising from *nkx6.1+* cells.

### *nkx6.1*-expressing cells are multipotent progenitors giving rise to all pancreatic cell lineages

**Fig. 2:**
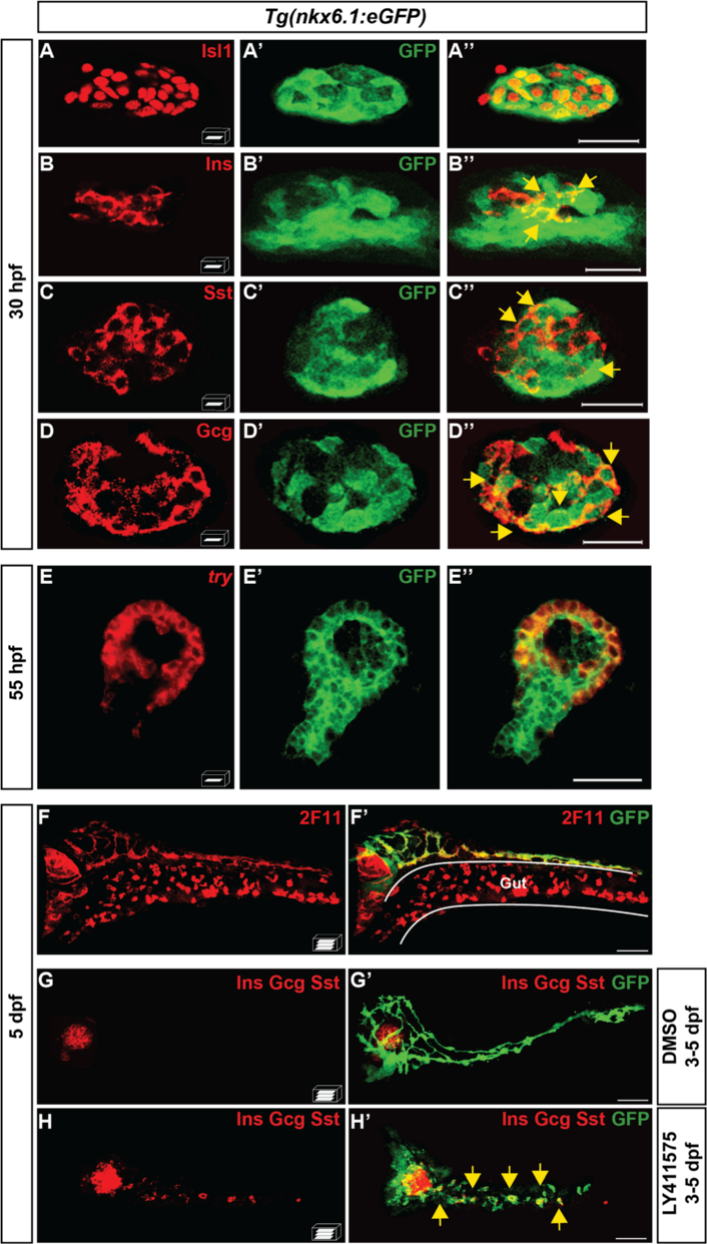
*nkx6.1*-expressing cells give rise to all pancreatic cell types. **a**–**d**" Immunodetection in 30-hpf *Tg(nkx6.1:eGFP)* embryos of GFP with Isl1 (**a**–**a**"), Ins (**b**–**b**"), Sst (**c**–**c**"), or Gcg (**d**–**d**"). *Yellow arrows* point to cells co-expressing GFP and the respective hormones. **e**–**e"** Fluorescent whole-mount in situ hybridization (WISH) of 55-hpf *Tg(nkx6.1:eGFP)* embryos using a *try* probe followed by GFP immunodetection. **f**, **f'** Immunodetection in 5-dpf *Tg(nkx6.1:eGFP)* embryos of GFP and of the hepato-pancreatic duct marker 2F11. **g**–**h'** Immunodetection of GFP and of Ins, Gcg, and Sst hormones in 5-dpf *Tg(nkx6.1:eGFP)* embryos treated from 3 to 5 dpf with dimethyl sulfoxide (DMSO) (**g**) or with the Notch signaling inhibitor, LY411575 (**h**). *Yellow arrows* point to secondary endocrine GFP+/hormones+ cells found in IPDs. All views are ventral with the anterior part to the left and represent either z-plane confocal images (**a**-**e**) or confocal projection images (**f**-**h**). Scale bars = 20 μm (**a**-**e**) or 40 μm (**f**-**h**)

Using short-term lineage tracing, we first assessed if *nkx6.1*+ cells can give rise to the first wave of endocrine cells of the dorsal bud using the *Isl1* marker, which labels all mature endocrine cells. In contrast to the endogenous *nkx6.1* and the *nkx6.1:GFP* transcripts, which are not co-expressed with *isl1* ([18] and Additional file 1: Fig. S1D–D"), GFP was detected in 40 ± 3.8 % of Isl1+ cells in *Tg(nkx6.1:eGFP)* embryos (*n* = 10) at 30 hpf indicating that *nkx6.1* cells can give rise to endocrine cells (Fig. 2a–a"). Also, we found GFP in all different endocrine cell types, i.e. in 35 ± 18.8 % of insulin+ (Ins+) cells (*n* = 21) (Fig. 2b–b''), 42 ± 12.5 % somatostatin+ (Sst+) cells (*n* = 4) (Fig. 2c–c"), and 77 ± 3.7 % of glucagon+ (Gcg+) cells (*n* = 5) (Fig. 2d–d"). The percentage of GFP+ cells in the different endocrine subtypes appeared to depend on the onset of expression for each hormone, which is from 15 hpf onward for *ins*, 17 hpf for *sst2* and 21 hpf for *gcga*. Therefore, when the first hormone-expressing cells differentiate from the pool of *nkx6.1*+ progenitors, only a minority of them have accumulated enough GFP to be detected, as explained above (see Fig. 1a and data not shown).

Next, we analyzed whether the *nkx6.1+* cells also contribute to the cells originating from the ventral bud (ductal, acinar, and secondary islets). With Nkx6.1 being expressed in all pancreatic ductal cells (as shown in Fig. 1e), we detected accordingly an expression of GFP in all pancreatic ducts labeled by 2F11 antibody (Fig. 2f, f'). In contrast, while endogenous *nkx6.1* never co-localizes with *trypsin* (data not shown), a marker of mature acinar cells, GFP was detected in a large majority of acinar cells at 55 hpf (70 ± 25 % of *trypsin*+ cells (*n* = 8)), the stage when acinar cells have just begun to differentiate (Fig. 2e, e'). Here again, the prolonged GFP detection in the acinar cells gradually disappears and, from 3 dpf, the acinar cells are no longer labeled with GFP (shown at 5 dpf in Fig. 2f'). And finally, to determine whether *nkx6.1+* cells can give rise to the secondary islets, emerging from IPDs, we treated *Tg(nkx6.1:eGFP)* larvae with the Notch-signaling inhibitor LY411575 from 3 to 5 dpf to increase the number of late endocrine cells and thereby facilitate their detection [7, 12, 16]. In LY411575-treated larvae, we observed an increase of the endocrine cells in the principal islet and the appearance of numerous endocrine cells in the pancreatic tail (*yellow arrows*, Fig. 2h, h'), as previously reported. All these endocrine cells are co-labeled with GFP (*n* = 4) indicating that the *nkx6.1+* ductal cells can also give rise to the secondary islets.

These data indicate that *nkx6.1*-expressing cells are multi-lineage pancreatic progenitors, which can differentiate into endocrine, acinar, and ductal cells.

### *ascl1b*-expressing cells give rise exclusively to the endocrine lineage

To determine whether *ascl1b* is expressed in the multipotent pancreatic progenitors or in the endocrine precursors, we determined the cell fate of the *ascl1b*-expressing cells. To that end, a BAC reporter *Tg(ascl1b:eGFP-2A-creER*^*T2*^*)* was engineered where the bicistronic transcript *eGFP-2A-creER*^*T2*^ is under the control of the promoter and regulatory sequences of *ascl1b*. Thus, we replaced the beginning of the *ascl1b* open reading frame with an *eGFP-2A-creER*^*T2*^ cassette (Additional file 2: Fig. S2A). The expression profile of GFP in the stable transgenic line *Tg(ascl1b:eGFP-2A-creER*^*T2*^*)* faithfully recapitulates the expression of the endogenous *ascl1b* transcript (Additional file 2: Fig. S2B–D).

**Fig. 3:**
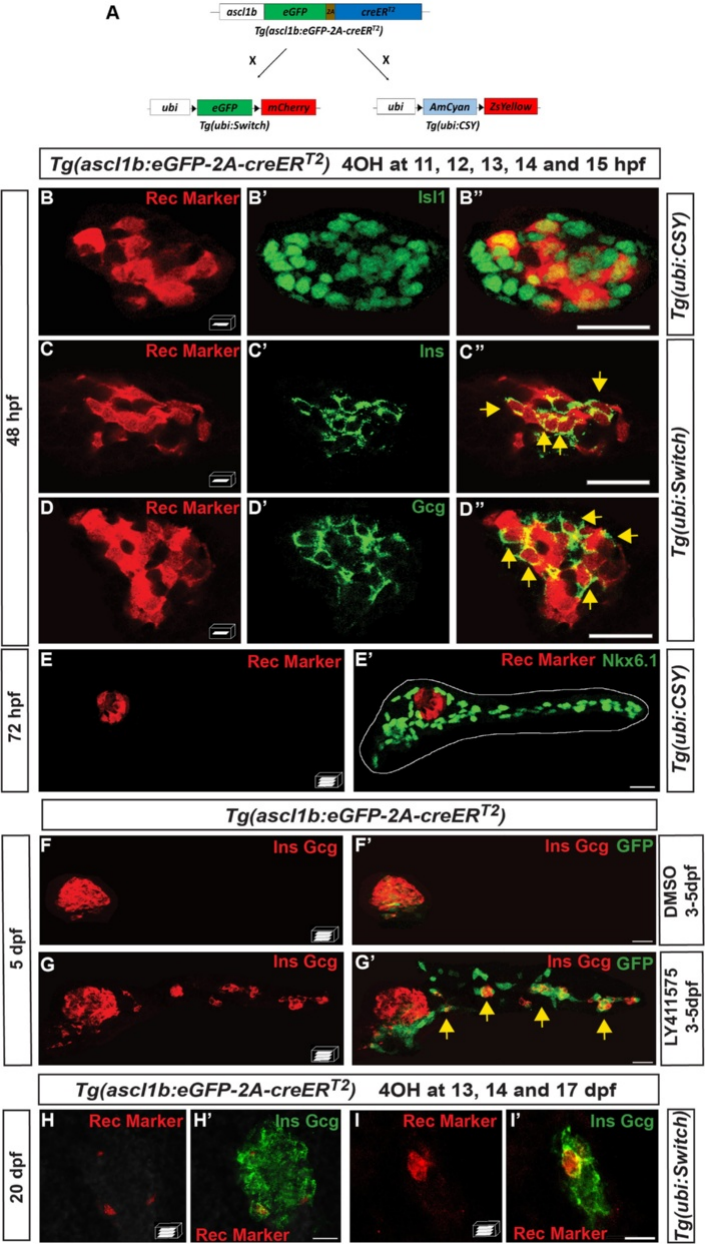
*ascl1b*-expressing cells give rise exclusively to endocrine cells of both dorsal and ventral bud. **a**–**e'**, **h**–**i'** Genetic lineage tracing using the *Cre-loxP system.* **a** Schematic representation of the genetic lineage tracing experiments. The transgenic line *Tg(ascl1b:eGFP-creER* ^*T2*^ *)* was crossed with the *Tg(ubi:loxP-eGFP-loxP-mCherry)* line, abbreviated *Tg(ubi:Switch)*, or with the *Tg(ubi:loxP-AmCyan-loxP-ZsYellow)* line, abbreviated *Tg(ubi:CSY)*, treated with 4-hydroxytamoxifen (4OHT) at 11, 12, 13, 14, and 15 hpf (**b**–**e'**) or at 13, 14, and 17 dpf (**h**–**i'**) and fixed for analysis at the indicated times. Black triangles in **a** represent loxP sites. **b**–**e'** Immunodetection of CRE-mediated rec markers (ZsYellow or mCherry, *red*) and Isl1 (**b**–**b**"), Ins (**c**–**c**"), Gcg (**d**–**d**"), or Nkx6.1 (**e**, **e'**) in 4OHT-treated embryos (*green*). The *dotted white line* delimits the pancreas (**e'**). *Yellow arrows* point to cells co-expressing rec marker (ZsYellow or mCherry) and the respective hormones (Ins or Gcg). **f**–**g'** Short-term lineage tracing: immunodetection of GFP and the Ins and Gcg hormones in 5-dpf *Tg(ascl1b:eGFP-creER* ^*T2*^ *)* embryos treated from 3 to 5 dpf with DMSO (**f**, **f'**) or with the Notch signaling inhibitor, LY411575 (**g**, **g'**). *Yellow arrows* in **g'** point to GFP+/Ins+/Gcg+ secondary endocrine cells found in the IPDs. **h**–**i'** Immunodetection at 20 dpf of the CRE-mediated rec marker mCherry together with Ins and Gcg in 4OHT-treated larvae. All views are ventral with the anterior part to the left and represent z-plane confocal images (**b**–**d**") or confocal projection images (**e**–**i'**). Scale bars = 20 μm

Cell fate experiments were performed with Cre/loxP-based lineage tracing approaches by crossing the *Tg(ascl1b:eGFP-2A-creER*^*T2*^*)* with Cre-responder transgenic lines, either *Tg(ubi:loxP-AmCyan-loxP-ZsYellow)*, termed *Tg(ubi:CSY)* [27], or *Tg(ubi:loxP-eGFP-LoxP-mCherry)*, termed *Tg(ubi:Switch)* [28] (Fig. 3a). The double-transgenic embryos were treated five times with 4-hydroxytamoxifen (4OHT) from 11 to 15 hpf, the period when *ascl1b* expression reaches its maximal level [15], and the embryos were analyzed at 48 or 72 hpf. With these five 4OHT treatments, many *ascl1b*-expressing cells have undergone CRE recombination while no recombination was detected in the treated single-transgenic embryos used as control (data not shown). The CRE-mediated recombination (rec) marker (standing for either ZsYellow or mCherry) analyzed in double-transgenic embryos at 48 hpf was detected in 38 ± 4.3 % of Isl1+ cells (*n* = 5), indicating that *ascl1b+* cells give rise to the endocrine cells of the dorsal bud (Fig. 3b–b"). In a similar way, the rec marker was detected in 58 ± 7.1 % of the Ins+ cells (*n* = 9) (Fig. 3c–c") and in 59 ± 3.7 % of the Gcg+ cells (*n* = 9) (Fig. 3d–d"). In contrast, the rec marker, clearly visible in the endocrine islet, was not detected at 72 hpf in the ductal cells, labeled by Nkx6.1, nor in the acinar cells, which surround them (Fig. 3e, e'), indicating that *ascl1b+* cells cannot give rise to exocrine cells. Finally, we determined whether the *ascl1b+* cells give rise to the secondary islets emerging from IPDs, either artificially induced by inhibiting the Notch pathway or naturally occurring in 20-dpf larvae. As the combined treatment of LY411575 with 4OHT was lethal, we performed short-term lineage tracing based on GFP expression (instead of Cre/loxP-based lineage tracing analyses). As shown in Fig. 3f, g, LY411575 treatment from 3 to 5 days of the *Tg(ascl1b:eGFP-2A-creER*^*T2*^*)* larvae led to the appearance of GFP cells all along the IPDs, most of these cells being also positive for glucagon or insulin hormones (*yellow arrows*, Fig. 3g, g'), indicating that *ascl1b*+ cells give rise to induced secondary islets. To trace the naturally occurring endocrine cells, we treated *Tg(ascl1b:eGFP-2A-creER*^*T2*^*); Tg(ubi:Switch)* larvae with 4OHT at 13, 14, and 17 dpf and analyzed the larvae at 20 dpf. The rec marker was detected within the principal islet (Fig. 3h, h') as well as in secondary islets (Fig. 3i, i') confirming that *ascl1b*+ cells can give rise to secondary islets.

In conclusion, our data demonstrate that *ascl1b+* cells exclusively give rise to the endocrine cells originating from both the dorsal and ventral buds.

### *nkx6.1* and *ascl1b* are first co-expressed in the endocrine precursors of the dorsal bud but rapidly their expression domain segregates

**Fig. 4:**
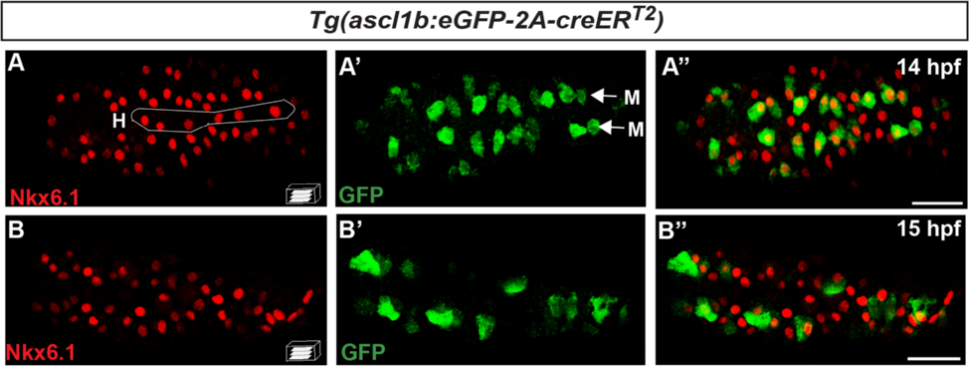
*nkx6.1* and *ascl1b* are first co-expressed in the endocrine precursors of the dorsal bud but rapidly their expression domain moves apart. Immunodetection of endogenous Nkx6.1 and GFP in *Tg(ascl1b:eGFP-creER* ^*T2*^ *)* embryos at 14 hpf (**a**–**a**") and 15 hpf (**b**–**b**"). All views are ventral with the anterior part to the left and represent confocal projection images. Scale bars = 40 μm. *H* hypochord, *M* medial cells

As presented above, *nkx6.1* is expressed in the multipotent pancreatic progenitors and *ascl1b* in the endocrine precursors; we therefore analyzed the relationship between these two populations by comparing the Nkx6.1 and GFP proteins in *Tg(ascl1b:eGFP-2A-creER*^*T2*^*)* embryos*.* At 14 hpf, the *ascl1b*:eGFP cells delineate two lines adjacent to the midline (Fig. 4a–a"). These cells correspond to the most medial endodermal cells (indicated by *M* in Fig. 4a'), reported to give rise mostly to pancreatic endocrine cells [29, 30]. At this stage, all of these *ascl1b*:eGFP cells also express Nkx6.1. In contrast, the Nkx6.1 expression domain is larger and, in addition to its expression in the hypochord (indicated by *H* in Fig. 4a), it is also expressed in the lateral cells, reported to give rise to exocrine and intestinal cells [29]. Rapidly, these two domains segregate since, as early as 1 hour later, the majority of *ascl1b:e*GFP+ cells no longer express Nkx6.1 (Fig. 4b–b"). Thus, separation of the two domains is largely completed when hormone-expressing cells start differentiating.

To determine whether such segregation results from a mutual repression, we examined whether the loss of *ascl1b* leads to an increase of *nkx6.1* expression and vice versa. We generated *ascl1b* and *nkx6.1* loss-of-function mutants using the CRISPR/cas9 genome editing technology [31] (see “Methods” and Additional file 3: Fig. S3A, A' and Additional file 4: Fig. S4A, A'). As shown in Additional file 3: Fig. S3, the loss of *ascl1b* does not affect the expression of *nkx6.1* (Additional file 3: Fig. S3D, E) while it significantly reduces the number of *sst2+* cells (Additional file 3: Fig. S3B, C), as reported for the *ascl1b* morphants [15]. In the same way, *ascl1b* expression does not increase in *nkx6.1* loss-of-function mutant embryos (Additional file 4: Fig. S4F, G), for which the effective loss of *nkx6.1* expression was confirmed by immunodetection of Nkx6.1 (Additional file 4: Fig. S4B, C) and by the drastic reduction in the number of Gcg+ cells (Additional file 4: Fig. S4D, E), as reported for the *nkx6.1* morphants [18].

### *ascl1b* and *nkx6.1* are regulated in an opposite way by the Notch signaling pathway

**Fig. 5:**
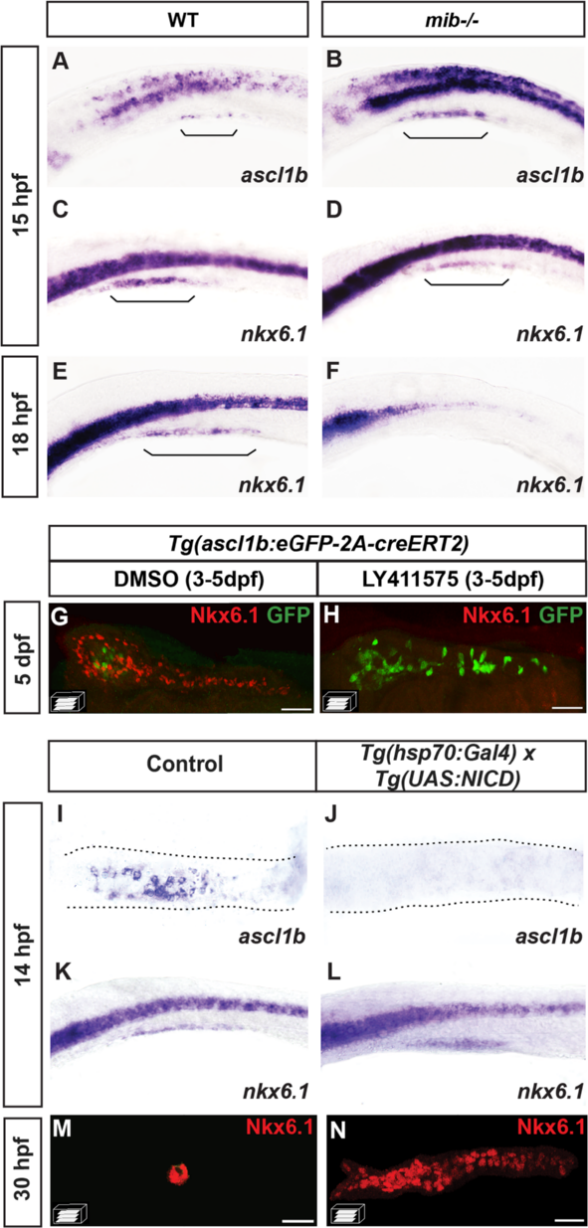
*ascl1b* and *nkx6.1* are regulated in an opposite way by the Notch signaling pathway. **a**–**f** WISH on wild-type (*WT*) embryos (**a**, **c**, **e**) or *mind bomb* (*mib* ^-/-^) mutants (**b**, **d**, **f**) with *ascl1b* (**a**, **b**) or *nkx6.1* (**c–f**) probes. *Brackets* delimit the pancreatic domain. **g**, **h** Immunodetection of GFP and endogenous Nkx6.1 in 5-dpf *Tg(ascl1b:eGFP-creER* ^*T2*^ *)* embryos treated from 3 to 5 dpf with DMSO (**g**) or with the Notch signaling inhibitor, LY411575 (**h**). **i**–**n** WISH with *ascl1b* (**i**, **j**) or *nkx6.1* (**k**, **l**) probes or immunodetection of endogenous Nkx6.1 (**m**, **n**) of double-transgenic *Tg(hsp70:Gal4)* x *Tg(UAS:NICD)* embryos (**j**, **l**, **n**) or of control simple-transgenic embryos (**i**, **k**, **m**), both heat-shocked at 11 hpf for 20 min. Lateral views (**a**–**f**, **k**, **l**) or ventral views (**g**–**j**, **m**, **n**) of embryos of the indicated stages with the anterior to the left. Scale bars = 40 μm

We then tested whether the segregation of the *ascl1b* and *nkx6.1* expression domains results from a different response to the Delta/Notch signaling pathway. To analyze the impact of Notch signaling on *nkx6.1* and *ascl1b* expression, we performed Notch loss- and gain-of- function analyses. We first analyzed the expression of *nkx6.1* and *ascl1b* in mind bomb mutants *(mib*^*ta52b*^) in which Notch signaling is disrupted [32]. As previously reported [15], expression of *ascl1b* is strongly increased in *mib* embryos at 15 hpf (Fig. 5a, b), in both the pancreas and the nervous system. The opposite effect was observed for *nkx6.1* whose expression is reduced at the same stage (Fig. 5c, d) and completely lost at 18 hpf (Fig. 5e, f). This suggests that the Notch signaling pathway is essential for maintaining *nkx6.1* expression but not for its initiation. This was confirmed by the finding that, at 13 hpf, *nkx6.1* expression was unchanged in *mib* mutants while *ascl1b* was already upregulated (Additional file 5: Fig. S5A–D).

The same conclusion was drawn for the ventral bud where treatment for 3–5 dpf with the LY411575 Notch inhibitor led to a complete loss of Nkx6.1 expression and a drastic increase of *ascl1b*:eGFP at 5 dpf (Fig. 5g, h). Like the dorsal bud, the initiation of *nkx6.1* expression in the ventral bud is not dependent on Notch signaling as its expression at 34 hpf was not perturbed in the *mib* mutant (Additional file 5: Fig. S5E, F).

For gain-of-function approaches, we crossed the *Tg(hsp70:Gal4)* with *Tg(UAS:NICD)* [33] and heat-shocked the embryos at 11 hpf to overexpress the Notch intracellular domain (NICD). At 3 hours after the heat-shock, we observed a complete loss of *ascl1b* expression (Fig. 5i, j) concomitant with an increase of *nkx6.1* in the NICD overexpressed embryos (Fig. 5k, l). This increase was even more important at 30 hpf, when a drastic expansion in the number of *nkx6.1+* cells was observed in the embryos overexpressing NICD (Fig. 5n) compared to the control (Fig. 5m).

In conclusion, these data show that Notch signaling represses *ascl1b* expression while it is essential for maintaining *nkx6.1* expression. By contrast, the initiation of *nkx6.1* expression is independent of Notch activity, both in the dorsal and the ventral buds.

### Most, but not all, Nkx6.1+ cells are Notch-responsive cells

**Fig. 6:**
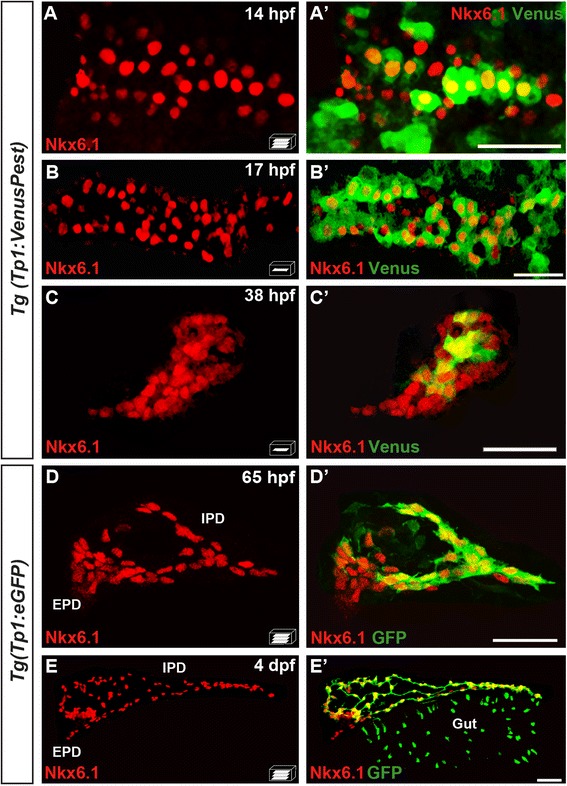
The pancreatic expression domain of Nkx6.1 includes the Notch-responsive cells. Immunodetection of endogenous Nkx6.1 (*red*) and Venus (revealed with anti-GFP, *green*) in *Tg(TP1:VenusPest)* (**a**–**c'**) or GFP in *Tg(Tp1:eGFP)* embryos (**d**–**e'**) at the indicated stages. All views are ventral with the anterior part to the left and represent either z-plane confocal images (**b**–**c'**) or confocal projection images (**a**, **a'**, **d**, **d'**, **e**, **e'**). Scale bars = 40 μm. *EPD* extra-pancreatic duct, *IPD* intra-pancreatic duct

*nkx6.1* being dependent on Notch signaling for maintaining its own expression, this prompted us to compare the location of the Notch-responsive cells and the Nkx6.1+ cells using the *Tg(Tp1:VenusPest)* [16] or *Tg(Tp1:eGFP)* [12] lines in which fluorescent markers are under the control of Notch-responsive elements (*Tp1*). In the prospective dorsal bud, we could detect Venus labeling in a subset of Nkx6.1 cells at 14 hpf (Fig. 6a, a') while 3 hours later (17 hpf), Venus was found in the vast majority of Nkx6.1+ cells (Fig. 6b, b'). Similarly, at the beginning of the formation of the ventral bud (38 hpf), only a subpopulation of Nkx6.1+ cells present some Notch activity (Fig. 6c, c') while at 65 hpf, the vast majority of Nkx6.1+ cells show Notch activity with the exception of the EPD anlagen (Fig. 6d, d'), known to be Notch inactive [34]. At 4 days, virtually all Nkx6.1+ cells are Notch-responsive in the IPDs (but the EPDs are still devoid of Notch activity) (Fig. 6e, e').

In conclusion, these observations indicate that, in both the dorsal and ventral buds, *nkx6.1+* cells progressively acquire Notch signaling activity, essential for maintaining *nkx6.1* expression.

### *nkx6.1* expression persists in ductal cells in the pancreas of adult zebrafish

**Fig. 7:**
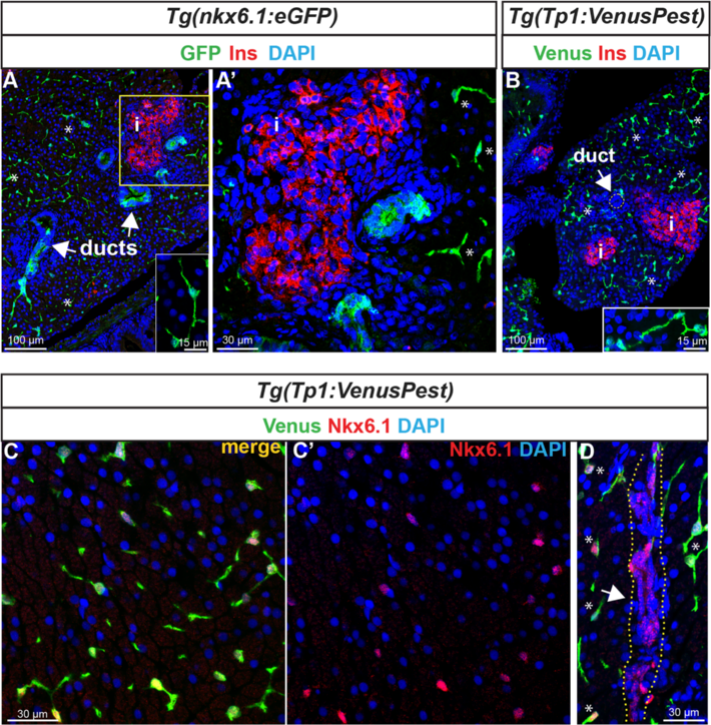
Expression of *nkx6.1* persists in duct cells in the adult pancreas. **a** GFP and insulin (Ins, *red*) immunodetection on section through the pancreas of adult *Tg(nkx6.1:eGFP)* zebrafish. *White arrows* point to pancreatic ducts and *asterisks* show cells (presumably centroacinar/ terminal end duct cells (CACs) dispersed throughout the exocrine tissue). **a'** Close-up of the islet highlighted with Ins. **b** Venus (*green*) and Ins (*red*) immunodetection in *Tg(Tp1:VenusPest)* showing the presence of Venus in CACs (*asterisks*) as previously reported [12], but not in duct cells within ductular structures (*white arrow*). Insets in (**a**) and (**b**) show isolated CACs. **c**–**d** Immunodetection of Venus (*green*) and of endogenous Nkx6.1 (*red*) in *Tg(Tp1:VenusPest)* revealing co-labeling of both markers in CACs (**c, c'**) while Nkx6.1 alone, but not Venus, labels the ducts. The *white arrow* in (**d**) points to a duct and *asterisks* indicate CACs. *Dotted yellow lines* delimit the duct. **c'** Same as (**c**) showing Nkx6.1 (*red*) and 4',6-diamidino-2-phenylindole (DAPI) only. *i*, islet

**Fig. 8:**
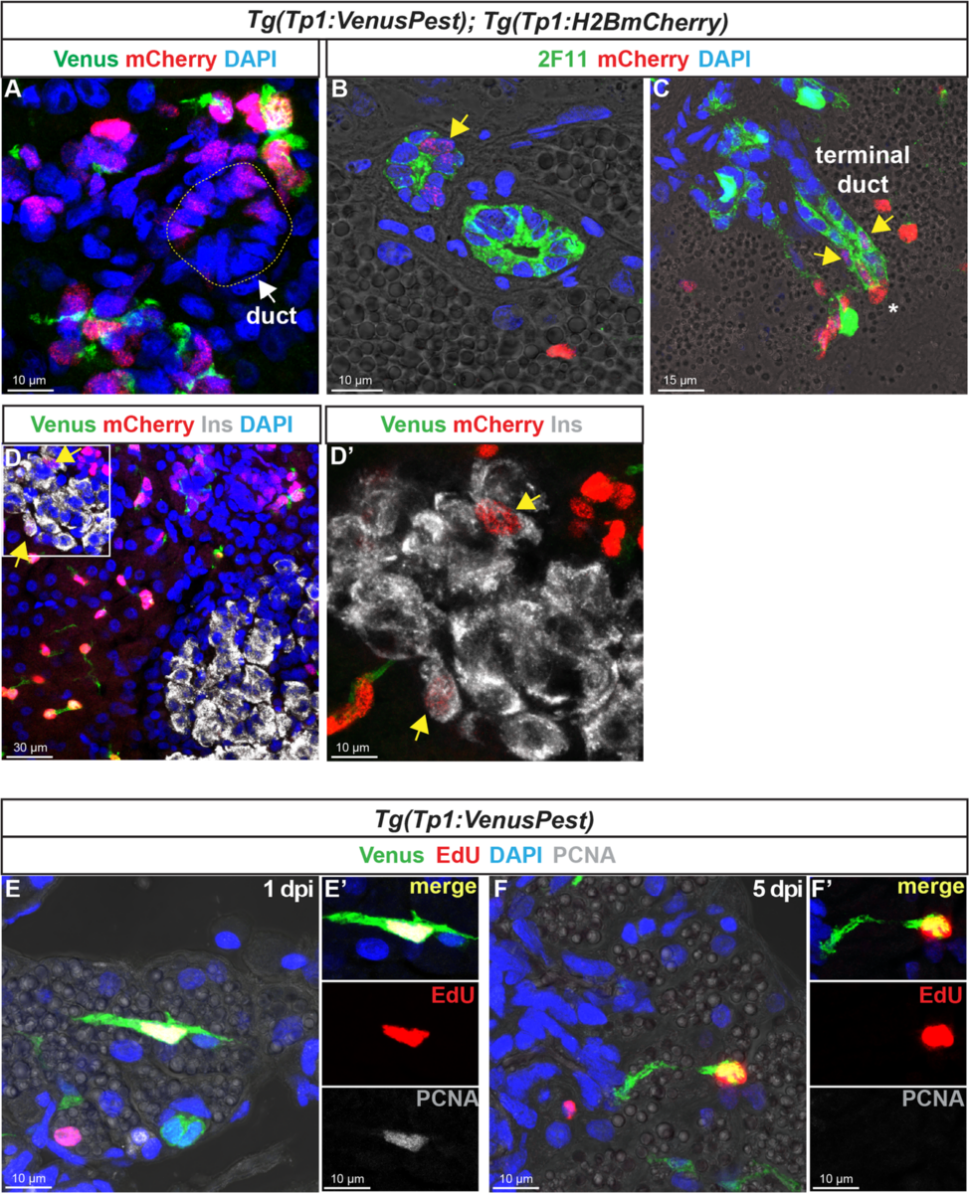
In the adult pancreas, Notch-responsive CACs give rise to other ductal and endocrine cells and have the capacity to replicate. Immunodetection on sections through the pancreas of adult *Tg(Tp1:VenusPest); Tg(Tp1:H2BmCherry).* **a** Comparison of Venus (*green*) and H2BmCherry (*red*) labeling showing a small duct containing H2BmCherry+ cells that have lost Venus. **b** Comparison of H2BmCherry (*red*) with the ductal marker 2F11 (*green*) showing some 2F11+ cells within a small duct co-expressing the stable H2BmCherry marker (*yellow arrow*). **c** Weak H2BmCherry labeling near the extremity of a ductular structure (terminal or intercalated duct) (*yellow arrows*); a CAC (intense H2BmCherry) at the tip of the terminal duct is indicated by an *asterisk*. **d** and **d'** (close-up) Some H2BmCherry+ cells, devoid of Venus (Notch off) co-express the beta cell marker Ins (*white*) (*yellow arrows*). Sections acquired in the head of the pancreas, at the level of the main endocrine islet. **e**–**f'** Detection of 5-ethynyl-2′-deoxyuridine (EdU) (*red*), Venus (*green*), and proliferating cell nuclear antigen (PCNA) (*white*) in *Tg(Tp1:VenusPest)* adult fish injected with EdU. The pancreas was analyzed 20 hours (1 day post injection) (**e**, **e'**) and 5 days (**f**, **f'**) after EdU injection

We then wanted to characterize *nkx6.1* expression in the pancreas of adult zebrafish. Immunodetection on paraffin sections through the pancreas of *Tg(nkx6.1:eGFP)* fish that were 6 to 9 months old revealed that *nkx6.1*:eGFP expression persists in adult zebrafish (Fig. 7a). Comparison of endogenous Nkx6.1 protein and GFP shows that, in the adult too, *Tg(nkx6.1:eGFP)* recapitulates the pattern of Nkx6.1 expression (data not shown). *nkx6.1*:eGFP expression is confined to the ducts and to isolated cells scattered throughout the exocrine tissue and was not detected in beta cells (Fig. 7a, a') or acinar cells. These *nkx6.1*:eGFP+ cells dispersed within the exocrine pancreas exhibit long cellular extensions characteristic of CACs (inset in Fig. 7a) [12]. In the adult zebrafish pancreas, as in mammals, the CACs can also be identified by Notch signaling activity [12]. Thus, to confirm the expression of *nkx6.1* in CACs, we used the Notch reporter line *Tg(Tp1:VenusPest)* [16], in which the destabilized Venus fluorescent protein (VenusPest) highlights cells harboring ongoing Notch activity, and which labels the CACs, as expected (Fig. 7b and inset). All Venus+ cells in the pancreas were found exclusively in the ductal system, and more particularly in the CACs and not within the ductular structures (Additional file 6: Fig. S6). Comparison of Venus with Nkx6.1 confirmed that Nkx6.1 is indeed expressed in all Venus+ CACs (Fig. 7c, c'). In contrast, while ducts also display *nkx6.1* expression as revealed with either *nkx6.1*:eGFP (Fig. 7a, a') or with the endogenous Nkx6.1 protein (Fig. 7d), they are devoid of Notch ongoing activity (*white arrows* pointing at Venus– ducts in Fig. 7b, d; see also Fig. 8a and Additional file 6: Fig. S6).

### Adult centroacinar cells display progenitor capacity in physiological conditions

We then asked whether CACs could generate other pancreatic cell types in adult zebrafish under physiological conditions by using the double *Tg(Tp1:VenusPest); Tg(Tp1:H2BmCherry*) in which the stable H2BmCherry protein labels cells harboring ongoing Notch activity (Venus+ mCherry+) and cells having previously experienced Notch activity (Venus– mCherry+) [16]. This tool has been previously exploited to characterize and follow the fate of the Notch-responsive progenitors in the IPDs of larvae [16, 35]. We thus characterized the Venus– H2BmCherry+ cells in adult fish to identify the pancreatic cell types derived from CACs. In about 30 % of all H2BmCherry+ cells, Notch activity was switched off (Venus–). Many of these cells, with weak H2BmCherry labeling, were identified within small ducts (Fig. 8a–c), which can be identified by the ductal marker 2F11 (Fig. 8b, c and Additional file 7: Fig. S7A-C for the separated colors and additional example) [11, 22, 36] or by Nkx6.1 (Additional file 7: Fig. S7D), and at the tip of which reside CACs (intense H2BmCherry, *asterisks* in Fig. 8c and Additional file 7: Fig. S7). Furthermore, low levels of H2BmCherry were also identified in some insulin-expressing cells (Fig. 8d, d'). About 4.9 ± 2.6 % (*n* = 4) of the H2BmCherry+/Notch off (Venus–) display Ins labeling. These findings show that mCherry+ terminal end duct cells and insulin-expressing cells originate from Notch positive CACs. Overall, this reveals that a subset of the Nkx6.1+ ductal cells, the Notch-responsive CACs, can generate ductal and endocrine cells.

To determine the capacity of CACs to replicate, their proliferative status was analyzed using 5-ethynyl-2′-deoxyuridine (EdU) labeling and proliferating cell nuclear antigen (PCNA) immunodetection in *Tg(Tp1:VenusPest)* adult fish. One day after EdU injection, a small fraction of CACs (5.8 ± 2.6 %, out of 600 counted CACs) had incorporated EdU. All these EdU+ Venus+ cells also express the proliferation marker PCNA (Fig. 8e, e'). In contrast, 5 days post injection, Venus+ cells that still display EdU labeling were no longer PCNA+ (Fig. 8f, f'), but still harbor characteristics of CACs, suggesting that they became post-mitotic CACs. The capacity of CACs to replicate together with their ability to undergo ductal and endocrine differentiation indicate that they can behave as adult pancreatic progenitor/stem cells in vivo.

### *nkx6.1*-expressing ductal cells contribute to beta cell regeneration in adult zebrafish

**Fig. 9:**
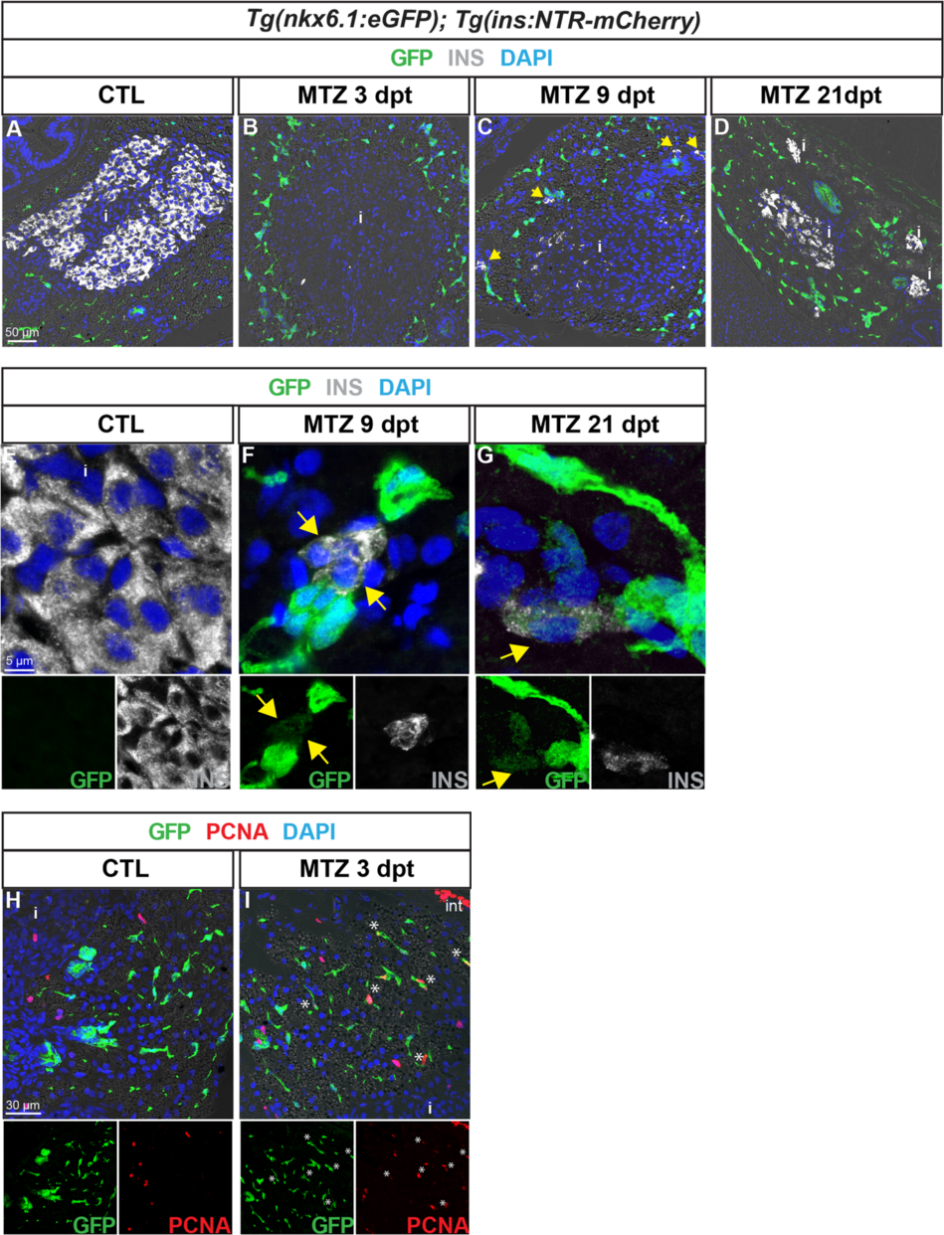
nkx6.1:eGFP+ cells proliferate and differentiate into new insulin-expressing cells after beta cell specific ablation. GFP (*green*) and Ins (*white*) labeling in the pancreas of *Tg(nkx6.1:eGFP); Tg(ins:NTR-mCherry)* adult fish. **a**, **b** Non-treated fish (*CTL*, **a**) show intense Ins staining in beta cells, while Ins+ cells are not detected 3 dpt with MTZ, indicating efficient ablation (**b**). Note that debris of one beta cell (Ins+) is observed in the islet. **c** At 9 dpt, Ins-expressing cells start to be detected in the principal islet and in extra-insular locations close to ductal GFP+ cells (*yellow arrows*). **d** At 21 dpt, islets show intense Ins staining consistent with beta cell recovery. **e**–**g** (and separate channels) While GFP is never detected in beta cells of control fish (**e**), some regenerating Ins+ cells display weak GFP labeling at both stages of regeneration analyzed, i.e. at 9 dpt (**f**) and 21 dpt (**g**). **h**, **i** (and separate channels) Beta cell ablation triggers proliferation of CACs as shown at 3 dpt (**h**) compared to CTL (**i**) (*asterisks*). *i* islet

The capacity of CACs to differentiate into beta cell in the normal adult zebrafish raises the question whether similar plasticity exists for regeneration. To induce beta cell regeneration, we used the *Tg(ins:NTR-mCherry)* line [1] and treatment with metronidazole (MTZ). The metronidazole is converted into a cytotoxic compound by the nitroreductase enzyme (NTR), which thereby triggers selective beta cell death by apoptosis [3]. In adult zebrafish, the ablation of beta cells causes dramatic hyperglycemia within 3 days rapidly followed by spontaneous normalization within 2 weeks and beta cell regeneration [2]. To analyze the ductal cells in the setting of beta cell regeneration, *Tg(nkx6.1:eGFP); Tg(ins:NTR-mCherry)* adult fish were treated with MTZ (day 1) and sacrificed at different time points during regeneration to analyze GFP and Ins on tissue sections at the level of the principal islet. The blood glucose level was measured just before sacrifice. At 3 days post treatment (dpt), ablation was total and effective as reported [2, 37]. MTZ-treated fish displayed severe hyperglycemia as expected (>500 mg/dl, *n* = 10 fish) while the glycemia of non-treated fish was at normal values of 61 ± 19 mg/dl (*n* = 11). Immunolabeling at 3 dpt indicated total beta cell ablation as manifested by the absence of insulin staining in the pancreas of MTZ-treated *Tg(nkx6.1:eGFP); Tg(ins:NTR-mCherry)* fish (Fig. 9a, b). At 9 dpt, the first new beta cells have started to reappear (Fig. 9c). Glycemia was still above normal values but nonetheless had decreased (145 ± 31 mg/dl, *n* = 2). The principal islet in the treated fish still showed very weak insulin expression but a few new beta cells (about 5 % of the islet cells, compared to 40–70 % in control fish) can be detected in the principal islet as well as throughout the exocrine tissue as isolated cells or as small clusters of Ins-expressing cells next to CACs and ductal cells marked by GFP (Fig. 9c, *yellow arrows*). The presence of insulin-expressing cells at 9 dpt is indicative of beta cell regeneration. Interestingly, some of the regenerating insulin+ cells displayed weak GFP staining (7.9 ± 3.2 % of the Ins+ cells, 452 counted cells, *n* = 2 fish) (Fig. 9f). These cells were found next to strongly GFP-labeled ductal nkx6.1:eGFP cells. GFP+ Ins+ cells were also detected at 21 dpt (11.1 ± 4.3 % of the Ins+ cells, 940 counted cells, *n* = 2 fish) (Fig. 9g). At this stage, a large number of beta cells have recovered (about 30–50 % of islet cells, Fig. 9d) [2, 37] and glycemia was normalized (57 ± 9 mg/dl). In contrast, insulin+ cells never harbor GFP labeling in non-treated fish (Fig. 9e). Thus, by using the same approach of short-term tracing of nkx6.1:eGFP+ cells as in embryos, this finding strongly suggests that ductal nkx6.1:eGFP+ cells contribute to regenerated beta cells.

Next we assessed proliferation in *Tg(nkx6.1:eGFP); Tg(ins:NTR-mCherry)* in response to beta cell ablation. Three days post treatment, *nkx6.1*:eGFP ductal cells showed increased proliferation as illustrated with PCNA (Fig. 9h, i). This was observed not only within ductal structures as previously described ([2] and not shown) but also for CACs. These observations suggest that ductal cells with pancreatic progenitor properties activate proliferation prior to differentiation into beta cells during regeneration.

### Transcriptomic analysis of adult *nkx6.1*+ pancreatic ductal cells

**Fig. 10:**
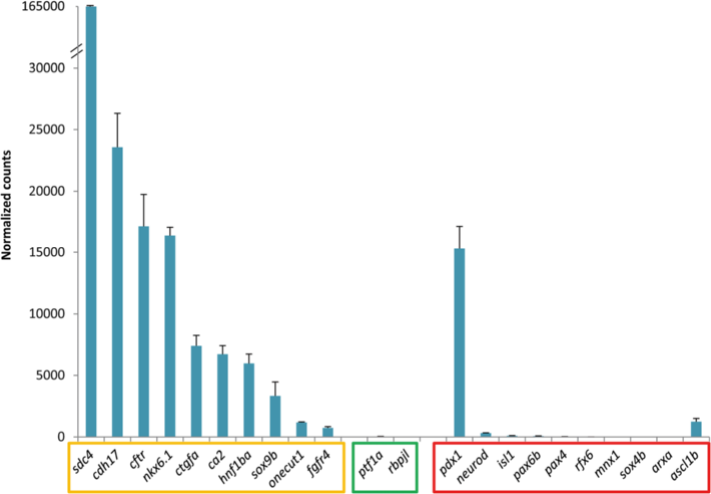
Expression of known pancreatic markers in the transcriptome of adult *nkx6.1*:eGFP cells. Expression values are expressed as normalized counts. High expression of known ductal genes (*orange box*) is detected while acinar (*green box*) and endocrine (*red box*) markers display low abundance, with the exception of *pdx1* and *ascl1b* (see text)

To get a comprehensive characterization of pancreatic *nkx6.1*+ ductal cells in adult zebrafish, we determined their transcriptome landscape. Ductal GFP+ cells were isolated from dissected pancreases of adult *Tg(nkx6.1:eGFP)* fish (with ~95 % purity) and used in RNA-seq experiments. The expression of 15,888 genes could be detected in the ductal transcriptome (complete data available at [38]), in which many genes already known to be expressed in pancreatic ducts in either zebrafish or mammals were found at high expression level, such as *sox9b*, *hnf1ba*, *onecut1/hnf6*, *cftr*, *cdh17*, *ca2*, and *ctgfa* in addition to *nkx6.1* (Fig. 10). We also detected expression of *fgfr4* and *sdc4*, recently proposed as novel ductal markers in the murine embryonic pancreas [39]*.* In contrast to these ductal genes, the acinar markers *ptf1a* and *rbpjl*, the pan-endocrine markers *pax6b* and *isl1*, and the lineage specific genes *mnx1* and *arx* were either not detected or detected at extremely low levels in the *nkx6.1:*eGFP+ ductal cells transcriptome (Fig. 10), underscoring the accuracy of our fluorescence-activated cell sorting (FACS) cell preparations. In contrast to mouse or human adults in which the embryonic pancreatic progenitor marker *Pdx1* is not expressed in the pancreatic ducts in normal condition, *nkx6.1:*eGFP+ duct cells of healthy zebrafish display a robust expression of *pdx1*. Comparison of the duct transcriptome with those of pancreatic acinar and endocrine cells (manuscript in preparation) highlighted 3,684 genes with preferential expression in duct cells. Among them, 293 duct-specific genes were identified with strong enrichment (≥16-fold) and low expression in the other pancreatic cells (Additional file 8: Table S1), in which we find *sox9b*, *onecut1/hnf6*, *cdh17*, *ctgfa*, and *nkx6.1*, corroborating their status as duct-specific markers. Various components of the Notch signaling pathway could also be identified, namely *notch2*, and different *Hairy and enhancer of split*-related genes (*her6*, *her9*, and *her15.1*), confirming that a subpopulation of *nkx6.1*+ cells (the CACs) experiences Notch activity. In addition to genes involved in the Notch signaling cascade, our analyses also identified novel duct-specific markers such as *id2a*, encoding for a HLH transcription factor, which we also detected co-expressed with *sox9b* by WISH in 3-dpf larvae (Additional file 9: Fig. S8), and several components of the Wnt pathways such as the Wnt and SFRP ligands *sfrp5*, *sfrp3/frzb*, and *wnt7bb* (Additional file 8: Table S1). At a lower expression level, we could also detect the Wnt receptor *fzd7a*, which was the only Wnt receptor significantly expressed in the adult pancreas (565 ± 106 normalized counts with 68-fold enrichment in the ducts). These observations suggest that Wnt signaling may play an important role in adult ducts.

Strikingly, the only endocrine transcription factor that displayed substantial expression (>1000 counts) and enrichment in the ductal transcriptome was the pro-endocrine gene *ascl1b* (Fig. 10). As *ascl1b* specifically marks the endocrine precursors during pancreas development, these data support our observation that, within the *nkx6.1:*eGFP+ cell domain, some cells activate a pro-endocrine differentiation program in normal adult zebrafish.

## Discussion

In this study, we determined during development the fate of two pancreatic cell populations marked by *nkx6.1* and *ascl1b* and found that *nkx6.1+* cells are bona fide multipotent pancreatic progenitors while *ascl1b+* cells represent committed endocrine precursors. We found also that *nkx6.1* is maintained in adult zebrafish in ducts and CAC/terminal end duct cells, which we show still have the potential to give rise to endocrine cells in normal (non-diabetic) animals. The progenitor potency of adult *nkx6.1+* cells is also reflected in their transcriptome through the expression of several pancreatic progenitor markers and by their capacity to generate new beta cells after beta cell ablation.

Although *ascl1b* marks the endocrine precursor cells, as opposed to *nkx6.1*, which is the first multipotent pancreatic progenitor marker known to date, it is surprising to note that this transcription factor begins to be expressed in the prospective pancreatic region at 10 hpf [15], i.e. more than 1 hour before the appearance of *nkx6.1* (11.5 hpf) [18]. *Pdx1*, known in the mouse to be also expressed in the multipotent pancreatic progenitors, appears even later (14 hpf [6]). This brings the interesting concept that the first cells in the pancreatic anlagen acquire an endocrine identity before acquiring a pancreatic identity, suggesting that the mechanisms controlling pancreatic and endocrine identity are not necessary linked and can act in parallel. This situation seems to be restricted to the dorsal bud as, later, *nkx6.1* is first expressed during the formation of the ventral bud and *ascl1b* is then detected in endocrine committed cells. This peculiar situation could be related to the different lineage potential of the dorsal versus ventral bud cells. Indeed the dorsal bud gives rise exclusively to endocrine cells while the ventral bud is able to give rise to all pancreatic cell types [4, 40].

After a transient overlapping expression in the dorsal pancreatic anlagen, *nkx6.1*- and *ascl1b*-expressing domains segregate progressively. Cross-repressive interactions between lineage-determining transcription factors have been proposed as a molecular mechanism for establishing lineage allocation in several tissues [41–43]. We show that such a mechanism does not occur here, as we could not observe any cross-repression between *nkx6.1* and *ascl1b* in the dorsal bud when analyzing both mutants. The segregation can instead be explained by the opposite effects of the Notch signaling pathway on *nkx6.1* and *ascl1b* expression. By loss- and gain-of-function experiments, we definitely prove that *ascl1b* is repressed, while *nkx6.1* is maintained, by Notch signaling in both the dorsal and ventral buds. The importance of Notch signaling in regulating *nkx6.1* expression has been also shown in mice where disruption of Notch signaling results in the loss of the pro-trunk determinant *Nkx6.1* and the acquisition of pro-acinar identity [44]. Direct binding of RBPJ-K to the *Nkx6.1* promoter supports a direct role of Notch signaling in the expression of *Nkx6.1*. In contrast, the initiation of *nkx6.1* expression is independent of Notch signaling in both zebrafish pancreatic buds. This is in accordance with our findings that Nkx6.1+ cells progressively become Notch-responsive PNCs being found only in a subpopulation of the Nkx6.1+ cells at the beginning of the formation of both the dorsal and ventral buds. This could explain why the PNCs, found in a subdomain of the ventral bud at an early stage, can differentiate only into ductal and endocrine cells, while *nkx6.1-*expressing cells give rise to all pancreatic cell types. As *nkx6.1* and *ptf1a* are initially co-expressed in the ventral bud primordium ([20] and our unpublished data), we can hypothesize that the *nkx6.1+/ptf1a+/*Notch on cells give rise to the ductal and late endocrine cells while the *nkx6.1+/ptf1a+/*Notch off cells will give rise to the acinar cells. This model appears to contradict the data of Wang *et al.* [7] showing that the ventral bud primordium consists of two non-overlapping cell populations: a *ptf1*-expressing domain and a Notch-responsive progenitor core. It is possible that this discrepancy is due to the tools used to label the Notch-responsive cells: in our study, we used the *Tp1bglob:VenusPest* transgenic line allowing the detection of cells with current Notch activity, while Wang and collaborators used the *Tp1bglob:hgmb1-mCherry* line, which could show a delay in mCherry detection.

In adult zebrafish, *nkx6.1* expression persists in the pancreas where it is specifically restricted to the ducts. This situation is different in the mouse as *Nkx6.1* persists in beta cells but not in adult ducts. In zebrafish, it is *nkx6.2*, an *nkx6.1* homolog with functional equivalence [18, 45], which is expressed specifically in beta cells [18], suggesting that the Nkx6 function in beta cells is fulfilled by Nkx6.2 in zebrafish. Persistence of *nkx6.1* expression in the zebrafish ducts is also associated with persistence of another pancreatic progenitor marker, *pdx1*, whose expression is also restricted to beta cells in mammals. Combined with other hallmarks of embryonic pancreatic progenitors such as *sox9b*, *hnf1ba*, and Notch signaling components, the expression of genes *nkx6.1* and *pdx1* suggests that at least some ductal cells behave as pancreatic progenitors in adult zebrafish. Moreover, detection of the endocrine precursor marker *ascl1b* in the adult *nkx6.1:e*GFP+ ductal cell transcriptome is consistent with some ductal cells initiating an endocrine differentiation program and transiently expressing *ascl1b*, even in physiological conditions. The progenitor potential of ductal cells is fully revealed in the setting of beta cell regeneration where *nkx6.1:e*GFP+ cells show increased proliferation as well as the ability to differentiate into new beta cells. The adult pancreatic progenitors contributing to beta cell regeneration could be the CACs, as supported by our observation that they are able to replicate and to generate other ductal cells as well as endocrine beta cells. On the other hand, we cannot exclude the possibility that other ductal cells also contribute to regeneration based on *Tg(nkx6.1*:eGFP), which labels more broadly the ducts. Nevertheless, although the tool we used here to monitor lineage tracing presents some limitations (based on the persistence of GFP in assessing the short-term lineage tracing of *nkx6.1*-expressing cells), during the revision of our manuscript, a study by Delaspre *et al*. [37] established through CRE-based genetic labeling that Notch-responsive cells give rise to regenerated beta cells in adult zebrafish. Our data are in full accordance with their findings, and support the conclusion that ductal cells, possibly CACs, possess regenerative capacity. To determine whether CACs only or other ductal cells contribute to beta cell regeneration will require other genetic lineage tracings with markers expressed only in ductular structures and not in CACs.

In contrast to mammals, the adult zebrafish has the remarkable capacity to regenerate its beta cells rapidly and spontaneously following their selective destruction [2]. Our findings showing that ductal cells, such as CACs, behave as pancreatic progenitors/stem cells in normal (non-diabetic) adult animals and during regeneration strongly suggest that they could also constitute a source of regenerated beta cells in diabetic mammalian models. In the mouse, adult murine CACs display endocrine and exocrine progenitor potential in vitro with self-renewing ability [46], but evidences of their potential in vivo as progenitors of endocrine cells are missing. Indeed, CRE-based cell tracing of *Hes1*+ terminal duct cells/CACs in adult mice failed to show any islet progenitor capacity while these cells seemed to contribute to the ductal tree [47]. One explanation for this difference would be that while zebrafish CACs present pancreatic progenitor activity, mammalian CACs have retained a very limited capacity in vivo, which could not be evidenced in the mouse model of beta cell regeneration used. Another explanation would be that CACs form a heterogeneous cell population. This latter hypothesis could be verified by the analysis of the expression of different ductal markers identified in our transcriptome, which could help determine the existence of different ductal cell subpopulations.

A perspective of our work would be to examine thoroughly in zebrafish ductal cells in physiological conditions and during beta regeneration to identify mechanisms that could then be harnessed to promote beta cell regeneration in mammals. A first clue is provided by the fact that *Nkx6.1* and *Pdx1* are normally not expressed in mammalian duct cells, in contrast to zebrafish. It is therefore tempting to hypothesize that inducing the expression of these two factors in the pancreatic ducts in the adult mouse or human could enhance their progenitor potential. Consistent with this hypothesis, driving ectopic *Pdx1* expression is already harnessed for transdifferentiating different cells into beta cells in vivo (liver cells, pancreatic acinar cells, …) [48–51] and for transdifferentiating pancreatic duct cells into beta cells in vitro [52]. Interestingly, the ability of *Pdx1* to reprogram liver cells into beta cells is substantially increased by the combined action of *Nkx6.1* [53], which potentiates induction of the early pancreatic master genes *Neurog3* and *Isl1*.

In the zebrafish larvae, two sources have been described for the regeneration of beta cells: progenitor cells in the developing ducts [35] and alpha cells [54]. From our data in adults, we cannot rule out the contribution by other cell types than ductal cells. Indeed, in addition to increased proliferation of ductal cells, some islet cells show also increased proliferation after beta cell destruction ([2] and our data not shown) suggesting the involvement of other pancreatic, and possibly endocrine, cell types in regeneration. These possibilities remain to be validated in the adult zebrafish.

Our transcriptomic characterization of *nkx6.1*+ cells in normal non-regenerating adult zebrafish identified many of the ductal markers known throughout species, showing that many of these genes and their expression are conserved between zebrafish and mammals, as for example, different markers of Notch signaling [55–57], and *sdc4* and *fgfr4*. These latter two genes have been previously proposed as ductal markers in the mouse embryo [39]. Additional markers were also identified here, notably *id2a. Id2* has been shown to be expressed in ductal epithelial cells of IFNγNOD mice, a model of a regenerating pancreas harboring hyperplasic ducts, and to be involved in their expansion [58]. We demonstrate here its expression in ducts in larvae and in healthy adult pancreas, which may correlate with a role in development and in ductal constitutive homeostasis. Our findings also reveal that, besides the Notch pathway, ductal cells specifically express various components of Wnt signaling pathways. The expression of the *fzd7a* receptor, of two secreted Wnt antagonists, *sfrp5* and *frzb/sfrp3*, and of the agonist *wnt7bb* suggests a complex control of the activity of the Wnt pathway(s) in adult pancreatic ducts. Whether and how these different factors orchestrate pancreatic duct development, homeostasis, and function remains to be determined. Numerous cross-talks between the Notch and the Wnt/beta-catenin pathways occur during development, tissue homeostasis, and disease, notably by regulating the balance of stem cells and differentiated cells (reviewed in [59]). During pancreas development, beta-catenin controls the patterning of multipotent versus bipotent embryonic pancreatic progenitors in the mouse, in part, by inhibiting Notch signaling [60]. Our findings raise the question whether similar interactions shape the fate decision of progenitors/stem cells in the adult pancreas.

## Conclusions

We have developed transgenic tools enabling the characterization of *nkx6.1+* and *ascl1b+* progenitor cell populations and showed that, in the zebrafish embryo, *nkx6.1*+ cells are multipotent pancreatic progenitors, while *ascl1b*+ cells represent committed endocrine precursors. In adult zebrafish, *nkx6.1* expression persists exclusively in the ductal tree, notably in CACs. Transcriptomic profiling of adult *nkx6.1*+ ductal cells reveals hallmarks of embryonic pancreatic progenitors and identifies novel ductal markers. Our data also strongly suggest that adult zebrafish ductal cells, possibly CACs, possess regenerative capacity. Further characterization of ductal cells in this animal model should bring new insight into regeneration in mammals and open up new perspectives for anti-diabetic therapies.

## Methods

### Zebrafish maintenance, mutant and transgenic lines, and LY411575 treatment

Zebrafish (*Danio rerio*) were raised and cared for according to standard protocols [61]. All animal work has been conducted according to national guidelines and all animal experiments described herein were approved by the ethical committee of the University of Liège (protocol numbers 371, 1285, and 1662). Wild-type embryos from the AB strain were used and staged according to Kimmel [62]. Homozygous mind bomb mutants were obtained by mating heterozygous fish for the (mib^ta52b^) allele [63]. The following transgenic lines were used: *Tg(hsp70l:Gal4)1.5*^*kca4*^ abbreviated *Tg(hsp:Gal4)* and *Tg(UAS:myc-Notch1a-intra)*^*kca3*^ abbreviated *Tg(UAS:NICD)* [33], *Tg(Tp1bglob:eGFP)*^*um14*^ abbreviated *Tg(Tp1:eGFP)* [12], *Tg(TP1bglob:VenusPest)*^*S940*^ abbreviated *Tg(TP1:VenusPest)* [16], *Tg(Tp1bglob:H2BmCherry)*^*S939*^ abbreviated *Tg(Tp1:H2BmCherry)* [16], Tg(*ubi:loxP-EGFP-loxP-mCherry)* abbreviated *ubi:Switch* [28], *Tg(ubi:loxP-AmCyan-loxP-ZsYellow)* abbreviated *ubi:CSY* [27], *Tg(ins:NTR-mCherry)* [1], and *Tg(nkx6.1:eGFP); Tg(ins:NTR-mCherry)*.

The LY411575 treatment was performed by incubating the embryos during the indicated period with a 10-μm LY411575 solution (Medchemexpress), replaced every day.

### Generation of BAC transgenic lines

The polymerase chain reaction (PCR) primers used to generate the constructs are listed in Additional file 10: Table S2. The *BAC:nkx6.1* (Imagenes, DKEY-173 K2) DNA was introduced by electroporation into SW102 *E. coli* (derived from DY380) [64]. These bacteria contain the lambda prophage recombineering system and a galactose operon where the galactokinase gene (*galK*) has been deleted. The e*GFP* gene was inserted into exon 1 of *nkx6.1*, replacing the beginning of the *nkx6.1* open reading frame (amino acids (aa) 1 to 149) using a two-step positive and negative *galk* selection [25, 64, 65]. During the first step, the cassette containing the *galk* gene was amplified by PCR with the pair of primers O180F and O253R, containing at the 5′ end 50 bases identical to the *nkx6.1* sequence to allow homologous recombination and electroporated into the bacteria SW102 containing *BAC:nkx6.1*. Only recombinant bacteria are able to grow on minimal medium containing galactose as carbon source. During the second step, the *galK* gene was replaced by the e*GFP* gene. The *eGFP* cassette was amplified by PCR with the primers O186F and O256R containing at the 5′ end the same 50 bases identical to the *nkx6.1* sequence to allow homologous recombination and the *eGFP* sequences to anneal to the *eGFP* cassette. After electroporation, the bacteria were plated on minimal medium containing two-deoxy-D-galactose (DOG), a galactose analogue that after phosphorylation by GalK, becomes toxic. Only bacteria that have lost the galk gene survived on DOG-containing medium. To facilitate the insertion of the BAC in the genome of zebrafish, the *iTol2* cassette was also inserted into the backbone of *BAC:nkx6.1-eGFP* [24, 25, 65]. The *iTol2* cassette was amplified by PCR with the pair of primers O215F and O216R. The final construct *(BAC:nkx6.1-eGFP)* was purified with Nucleobond® BAC100 (Macherey-Nagel) and injected into the cytoplasm of one-cell-stage zebrafish embryos together with the mRNA for the transposase. The embryos and larvae were screened for GFP expression and the fluorescent injected fish were raised to adulthood and the offspring were screened for fluorescence. The transgenic line obtained was abbreviated to *Tg(nkx6.1:eGFP)* in the article.

To generate the *(ascl1b:eGFP-2A-creER*^*T2*^*)* transgenic line, we used *BAC:ascl1b* (Imagenes, DKEY-265 N18) spanning from 61 kb upstream and 89 kb downstream of the *ascl1b* gene. The *GFP-2A-creER*^*T2*^ cassette was inserted into exon 1 of *ascl1b*, replacing the beginning of the *ascl1b* open reading frame (aa 1 to 163) using the same two-step positive and negative *galk* selection as described above. For the first step, the cassette containing the *galk* gene was obtained by PCR using the primers O275F and O276R and for the second step, the *GFP-2A-creER*^*T2*^ cassette was amplified using the primers O277F and O278R.

### 4OHT treatment for creER^T2^ induction

4-Hydroxytamoxifen (4OHT, Sigma H7904) was dissolved in DMSO as a stock solution of 10 mM and kept in single-use aliquots in the dark at –70 °C. A working concentration of 10 μM 4OHT was demonstrated to lead optimally to Cre-mediated recombination without causing deleterious development defects. Embryos were treated five times from 11 to 15 hpf in E3 containing 10 μM 4OHT and kept in the dark at 28 °C. After the treatments, the embryos were washed in fresh E3 and fixed at 48 hpf or 72 hpf.

### CRISPR/cas9 genome mutagenesis

The *nkx6.1* and *ascl1b* mutant lines were generated by CRISPR/Cas9 technology essentially as described previously [31, 66]. The targeted sites were selected using the ZiFiT software package [67] in the first exon of *nkx6.1* (CCAAACCCCTGACAGAGCTTC) before the homeodomain coding region and in the first exon of *ascl1b* (GGAGACGCTGCGCTCCGCCGTGG) corresponding to the helix-loop-helix coding domain. The selected oligonucleotides were inserted into the plasmid DR274 (Addgene) and the gRNA (guide RNA) was synthesized by in vitro transcription using T7 RNA polymerase. Fertilized zebrafish eggs were injected with about 1 nl of a solution containing 50 ng of gRNA and 300 ng of nls-zCas9-nls mRNA obtained by transcription of the plasmid pT3TS-nCas9n (Addgene). The efficiency of mutagenesis was verified by genotyping using Heteroduplex Migration Assays [68] after amplification of targeted genomic sequences. Injected embryos were raised until adulthood and crossed with wild-type fish to generate heterozygote mutant F1 fish. Fish harboring frame-shift mutations were kept and used to raise F2 mutant lines, i.e. the *ascl1b*^*ulg-M2C*^ and *nkx6.1*^*ulg-M5*^ lines carrying, respectively, 11 and seven nucleotide deletions.

### Whole mount in situ hybridization, whole mount immunohistochemistry, and immunohistochemistry on paraffin sections

Double fluorescent and visible WISH were performed as previously described [69, 70] with the following probes: *ascl1b* [15, 71], *nkx6.1* [18, 72], *somatostatin 2 (sst2)* [73], and *try* [6].

Immunohistochemistry (IHC) on whole-mount embryos was performed as described [15]. For IHC on paraffin sections with adult tissues, adult fish between 6 and 9 months old were fixed in 4 % PFA (paraformaldehyde) overnight at 4° after euthanasia and opening of the abdominal skin. The digestive tract was then dissected and embedded in paraffin following standard procedures. Then, 5-μm sections were collected through the head of the pancreas at the level of the main pancreatic islet. Immunodetection was performed after standard antigen retrieval.

The antibodies used were: polyclonal rabbit anti-mCherry/dsRed (Living Colors DsRed Polyclonal Antibody, Clontech) 1:500, polyclonal rabbit anti-ZsYellow (Living Colors anti-RCFP polyclonal pan from Clontech) 1:300, chicken anti-GFP (Aves lab) 1:1000, mouse monoclonal anti-Nkx6.1 (clone F55A10) 1:20, mouse monoclonal anti-Isl1 (Hybridoma bank) 1:50, guinea pig anti-insulin (Dako) 1:500, mouse anti-glucagon (Sigma) 1:300, polyclonal rabbit anti-somatostatin (MP Biomedicals) 1:300, mouse anti-PCNA (Sigma) 1:1000, mouse 2F11 mAb (Abcam) 1:1000, and Alexa Fluor secondary antibodies (Invitrogen). Venus was detected with anti-GFP. Finally, 4',6-diamidino-2-phenylindole (DAPI) was used as nuclear staining.

Images were acquired with a Leica SP2 or SP5 confocal microscope and processed with Imaris 7.2.3 and Photoshop CS5. To count the Tp1:H2BmCherry+ cells expressing insulin, the total number of pancreatic mCherry+/Venus– and mCherry+/Ins+ cells was calculated for six sections every 15 μm for four fish.

### EdU injection and detection in adult zebrafish

A 12.5-mM EdU solution in DPBS (Dulbecco's Phosphate Buffer Saline) containing 0.25 % DMSO was injected intraperitoneally in 6–9-month-old fish at 100 μg/g body weight after anesthesia in tricaine methane sulfonate. Fish were then sacrificed and fixed in 4 % PFA. EdU (Click-iT® Labeling Technologies, Life Technologies) incorporation was detected on paraffin sections with Alexa Fluor555 before proceeding to IHC detection.

To count EdU+ cells, the total number of pancreatic Venus+ CAC and Venus+/EdU+ cells was calculated in three to six sections every 15 μm for four fish.

### Induction of beta cell ablation in adult zebrafish

Adult *Tg(nkx6.1:eGFP); Tg(ins:NTR-mCherry)* zebrafish between 6 and 9 months old were treated in fish water containing 10 mM MTZ (three to four fish per 500 ml, Sigma 3761) for 20 hours at 28 °C. Then the water was replaced twice before re-integration into the system.

Fish were anesthetized with tricaine and their glycemia was measured using the Accu-Chek Aviva glucometer system (Roche Diagnostics) with blood collected at the level of the tail. To minimize variations, the fish were fasted for 24 hours before measurement. After decapitation, the whole fish were fixed in 4 % PFA overnight at 4 °C. The digestive tract was then dissected prior to paraffin embedding.

### FACS purification of ductal cells

The pancreas of three to five *Tg(nkx6.1:eGFP*) adult fish (6–9 months old) were dissected and collected in HBSS with calcium. Dissociation was performed in HBSS (Hank's Balanced Salt Solution) with Ca^2+^/Mg^2+^ supplemented with 1 mg/ml collagenase IV (Life Technologies 17104-019) and collagenase P (Roche 1121386501) and 1.5 mg/ml dispase II (Life Technologies 17105-041) for 20 min at 30 °C. After several washes in HBSS without Ca^2+^/Mg^2+^, a single-cell suspension was obtained by Tryple Select 1× incubation for 10 min. Dissociation was stopped by HBSS without Ca^2+^/Mg^2+^ containing 1 % BSA (bovine serum albumin) and 2 mM EDTA (ethylenediaminetetraacetic acid). GFP+ cells were isolated on FACS Aria II under purity mode and the purity of the sorted cells was confirmed for a small fraction with an epifluorescence microscope (~95 %). Cells were immediately lyzed in 3.5 μl of reaction buffer (SMARTer Ultra Low RNA kit for Illumina sequencing, Clontech) and stored at –80 °C. Three independent replicates were generated from 6–9-month-old fish.

### cDNA synthesis and library preparation

cDNA synthesis was performed using the SMARTer Ultra Low RNA kit for Illumina sequencing (Clontech) according to the manufacturer’s recommendations. Then 3000–5000 sorted GFP+ cells were directly lyzed in 3.5 μl of reaction buffer and immediately frozen at –80 °C. cDNAs were synthesized, purified with Ampure XP beads and then amplified with 13 PCR cycles with Advantage 2 Polymerase Mix (50×, Clontech). The PCR products were purified on SPRI AMPure XP beads (Beckman Coulter), and the size distribution was checked on a high-sensitivity DNA chip (Agilent Bioanalyzer). cDNA libraries were prepared with TruSeq Nano DNA kit or Nextera XT DNA (Illumina). For TruSeq Nano libraries, 20–30 ng cDNA was sheared by sonication (parameters adjusted to obtain fragments from 350 to 450 bp). For Nextera libraries, 1 ng was fragmented by tagmentation. Then cDNA libraries were prepared according to the manufacturer’s recommendations. Samples were sequenced on an Illumina HiSeq 2000 at an average of 72.3 million 100-bp paired-end reads. RNA-seq data have been deposited in the European Nucleotide Archive from EMBL-EBI [38].

### Data analysis of the duct transcriptome

Before mapping, the first 30 bases of each read were trimmed to remove the adapters incorporated by the cDNA synthesis process. Trimmed reads were mapped to the zebrafish genome (Zv9, Ensembl genes version 75, ensembl.org) using the Tophat v.2.0.9 software [74]. For the three replicates, the total number of reads for Duct 1 was 40,336,250 (79.9 % mapped), for Duct 2 was 86,308,531 (82.5 % mapped), and for Duct 3 was 91,980,011 (67.6 % mapped).

HT-Seq count was used to estimate the expression level by counting how many reads align to each gene of the annotation (gene set, Ensembl.org) [75]. The expression of 15,888 genes was detected with at least one read in all three replicates.

To describe the set of genes enriched or preferentially expressed in ductal tissue, the ductal transcriptome was compared with acinar and endocrine transcriptomes prepared following the same methodology (composed of alpha, beta, and delta cells, not shown here, manuscript in preparation). The R package EBSeq was used to call differential expressed genes [76]. Ductal genes were identified based on their posterior probability (adjusted by false discovery rate) of being differentially expressed from the other two cell types. It was found that 3,684 genes were preferentially expressed in the duct transcriptome. Stricter thresholds were applied to identify genes with highly specific ductal expression (see “Results”).

## Additional files

Additional file 1: Figure S1. The bacterial artificial chromosome (BAC) reporter line *Tg(nkx6.1:eGFP)* mirrors the expression of the endogenous *nkx6.1* gene. **A** Schematic representation of the −55 to +95 kb *nkx6.1:eGFP* BAC transgene*.* This BAC includes sequence blocks that are highly conserved amongst vertebrates (*black boxes*) located from 11 to 77 kb downstream of the *nkx6.1* gene. By BAC recombineering using galK selection, the *eGFP* cassette (*green box*) was introduced into exon 1, replacing the beginning of the nkx6.1 open reading frame (aa 1 to 149). **B** Epifluorescence microscopy images of the immunodetection of endogenous Nkx6.1 (*red*) and GFP (*green*) in *Tg(nkx6.1:eGFP)* embryos. Lateral views of 48 hpf embryos with the anterior part to the left. **C** Confocal projection images of whole mount fluorescent in situ hybridization on a *Tg(nkx6.1:eGFP)* embryo showing that the *gfp* transcripts are not expressed in the insulin+ cells at 20 hpf. **D** Confocal projection images of whole mount fluorescent in situ hybridization on a *Tg(nkx6.1:eGFP)* embryo showing that the *gfp* transcripts are not expressed in the *isl1*+ cells at 30 hpf. Scale bar = 15 μm. *P* pancreas. (PNG 2609 kb) Additional file 2: Figure S2. The bacterial artificial chromosome (BAC) reporter line *Tg (ascl1b:eGFP-creER* ^*T2*^ *)* mirrors the expression of the endogenous *ascl1b* gene. **A** Schematic representation of the −61 to +89 kb *ascl1b:eGFP-2A-creER* ^*T2*^ BAC transgene. By BAC recombineering using galK selection, the *eGFP-2A-creER* ^*T2*^ cassette is introduced into exon 1, replacing the beginning of the *ascl1b* open reading frame (aa 1 to 163). **B** Epifluorescence microscopy images of the immunodetection of GFP in 17-hpf *Tg(ascl1b:eGFP-creER* ^*T2*^ *)* embryos. **C, D** Visible WISH showing expression of endogenous *ascl1b* in a wild-type (WT) embryo (**C**) and of *GFP* in *Tg(ascl1b:eGFP-creER* ^*T2*^ *)* embryos (**D**) at 15 hpf. Lateral views with the anterior part to the left. *P* pancreas. (PNG 1068 kb) Additional file 3: Figure S3.
*nkx6.1* expression is not repressed by Ascl1b. **A** Schematic representation of wild-type (WT) and mutant Ascl1b^ulg-M2C^ proteins. The basic domain (*b*) (+70/+78) is represented by a *blue box* and the helix loop helix (*HLH*) domain (+79/+123) by a *green box*. The coding region of the mutant Ascl1b^ulg-M2C^ protein contains an 11-bp deletion after the aa 107 codon, leading to a frameshift and the production of an aberrant region of 48 aa instead of the second helix, known to be essential for the function of the bHLH proteins. **A'** Table showing part of the nucleotide and protein sequence of the *ascl1b* gene and of the mutated form in the Ascl1b^ulg-M2C^ mutant. **B, C** WISH showing the drastic reduction of somatostatin expression in the ascl1b^-/-^ mutant compared to the WT embryo at 30 hpf. This phenotype is identical to the one of embryos injected with a translation-blocking morpholino, targeting the translation start site of ascl1b mRNA [15, 77, 78], suggesting that the mutant Ascl1b^ulg-M2C^ is effectively a null mutant. **D, E** Confocal projection images of Nkx6.1 immunodetection showing equivalent number of nkx6.1+ cells in WT and *ascl1b* ^-/-^ mutants at 16 hpf. All views are ventral with the anterior part to the left. Scale bars = 40 μm. (PNG 1321 kb) Additional file 4: Figure S4.
*ascl1b* expression is not repressed by Nkx6.1. **A** Schematic representation of wild-type (WT) and mutant Nkx6.1^ulg-M5^ proteins with the *yellow box* representing the homeodomain and the *red box* representing the NK domain. The coding region of the mutant Nkx6.1^ulg-M5^ protein contains a 7-bp deletion after the aa 141 codon, leading to the apparition of a STOP codon just after the deletion. The *black line* represents the region of the protein recognized by the Nkx6.1 antibody. **A'** Table showing part of the nucleotide and protein sequence of the nkx6.1 gene and of the mutated form in the Nkx6.1^ulg-M5^ mutant. **B, C** Nkx6.1 immunodetection showing the loss of the full-length Nkx6.1 protein in the *nkx6.1* ^*-/-*^ mutant. **D, E** Glucagon immunodetection showing a drastic reduction of the number of glucagon-expressing cells in the *nkx6.1* ^-/-^ mutant compared to the WT embryo at 30 hpf. This phenotype is the same as the one of embryos injected with the MO1 *translation*-blocking *morpholino*, targeting the translation start site of *nkx6.1* mRNA [18], shown to prevent *nkx6.1* expression efficiently in the neural tube [72]. This strongly suggests that the mutant Nkx6.1^ulg-M5^ is a null mutant. **F, G** Confocal projection images of fluorescent WISH showing equivalent number of *ascl1b*+ cells in WT and *nkx6.1* ^-/-^ mutants at 15 hpf. All views are ventral with the anterior part to the left. Scale bars = 40 μM. (PNG 1527 kb) Additional file 5: Figure S5. The initiation of *nkx6.1* expression is independent of Notch signaling. **A–D** Fluorescent WISH showing a drastic increase in the pancreatic dorsal bud of *ascl1b* expression in the *mind bomb (mib* ^*-/-*^ *)* mutants while *nkx6.1* expression is not affected at 13 hpf. **E, F** Fluorescent WISH showing that the pancreatic expression of *nkx6.1* in the ventral bud is not perturbed at 34 hpf in *mib* ^*-/-*^ mutants. The ventral (**A, B, E, F**) or lateral (**C, D**) views represent confocal projection images with the anterior to the left. Scale bars = 40 μM. *NS* nervous system. (PNG 968 kb) Additional file 6: Figure S6. All Notch-responsive cells in the adult pancreas are CACs. Notch active cells in *Tg(Tp1:VenusPest)* adult fish were labeled with anti-GFP to visualize VenusPest (*green*) and the ductal marker 2F11 (*red*). VenusPest labeling is detected throughout the exocrine tissue (see *asterisks* highlighting examples) and where it highlights the CACs together with 2F11. In contrast, ductal structures with intense 2F11 staining do not exhibit any Venus+ (Notch on) cells. Note also the presence of 2F11 in endocrine islet cells as previously reported [79]. (PNG 2854 kb) Additional file 7: Figure S7. Notch-responsive terminal end duct cells/CACs give rise to other ductal cells. Immunodetection of H2BmCherry and ductal markers in the pancreas of adult *Tg(Tp1:VenusPest); Tg(Tp1:H2BmCherry)* zebrafish*.* **A–C** H2BmCherry+ cells (*red*) in ductular structures labeled with the ductal (ducts and CACs) marker 2F11 (*green*). **A, B** separate channels of Fig. 8b–c. **B, C** Weak H2BmCherry labeling is present near the extremity of a terminal (or intercalated) duct (*yellow arrows*). The *asterisks* identify a CAC at the tip of the duct (strong H2BmCherry labeling). **D** Comparison of H2BmCherry (*red*) with endogenous Nkx6.1 (*green*) showing ductal Nkx6.1+ cells co-expressing H2BmCherry (*yellow arrows*). (PNG 4967 kb) Additional file 8: Table S1. Duct-specific genes listed by decreased expression level. We found 293 genes corresponding to the following “specificity” criteria: gene expression level normalized counts >1000 in duct and <1000 in non-ductal (averaged endocrine and acinar) transcriptome and ≥16 fold enrichment in ducts versus non-duct (averaged endocrine and acinar), expressed in log_2_ ratio (≥4). Ratio is mean of normalized counts in duct transcriptomes/mean of normalized counts in endocrine and in acinar transcriptomes. Known ductal markers and genes involved in the Notch pathway are highlighted in red. Novel markers and genes involved in the Wnt pathway are highlighted in green. (DOCX 26 kb) Additional file 9: Figure S8. Ductal expression of *id2a* during zebrafish development. Whole mount fluorescent in situ hybridization showing *id2a* (*green*) and the ductal marker *sox9b* (*red*) in a 3-dpf larva. The expression of both genes overlaps as highlighted by *yellow* labeling. In addition to expression in the intrapancreatic ducts (*IPD*), *id2a* is also expressed in the intrahepatic (*IHD*). *HPD* hepatopancreatic ducts. (PNG 537 kb) Additional file 10: Table S2. List of the primers used in this study. The 50 nucleotides added to allow homologous recombination are indicated in capital letters. (DOCX 12 kb)

## Abbreviations

4OHT: 4-hydroxytamoxifen
aa: amino acid
ARP: atonal related protein
ASCL: achaete-scute like
BAC: bacterial artificial chromosome
bHLH: basic helix-loop-helix
bp: base pair
CAC: centroacinar/terminal end duct cell
creER^T2^: 4OHT inducible Cre-recombinase
DMSO: dimethyl sulfoxide
dpf: days post fertilization
DOG: two-deoxy-D-galactose
dpt: days post treatment
EdU: 5-ethynyl-2′-deoxyuridine
eGFP: enhanced green fluorescent protein
EPD: extra-pancreatic duct
FACS: fluorescence-activated cell sorting
Gcg: glucagon
GFP: green fluorescent protein
hpf: hours post fertilization
IHC: immunohistochemistry
Ins: insulin
IPD: intra-pancreatic duct
kb: kilobyte
MTZ: metronidazole
NICD: Notch intracellular domain
NTR: nitroreductase enzyme
PCNA: proliferating cell nuclear antigen
PCR: polymerase chain reaction
PNC: pancreatic Notch-responsive cell
rec: recombinant
Sst: somatostatin
VenusPest: Venus fluorescent protein
WISH: whole-mount in situ hybridization
WT: wild type
