# Supplementary figures and images for: Progenitor potential of nkx6.1-expressing cells throughout zebrafish life and during beta cell regeneration
Source: BMC Biol. 2015 Sep 2;13:70. doi: 10.1186/s12915-015-0179-4 (PMC4556004; doi:10.1186/s12915-015-0179-4)

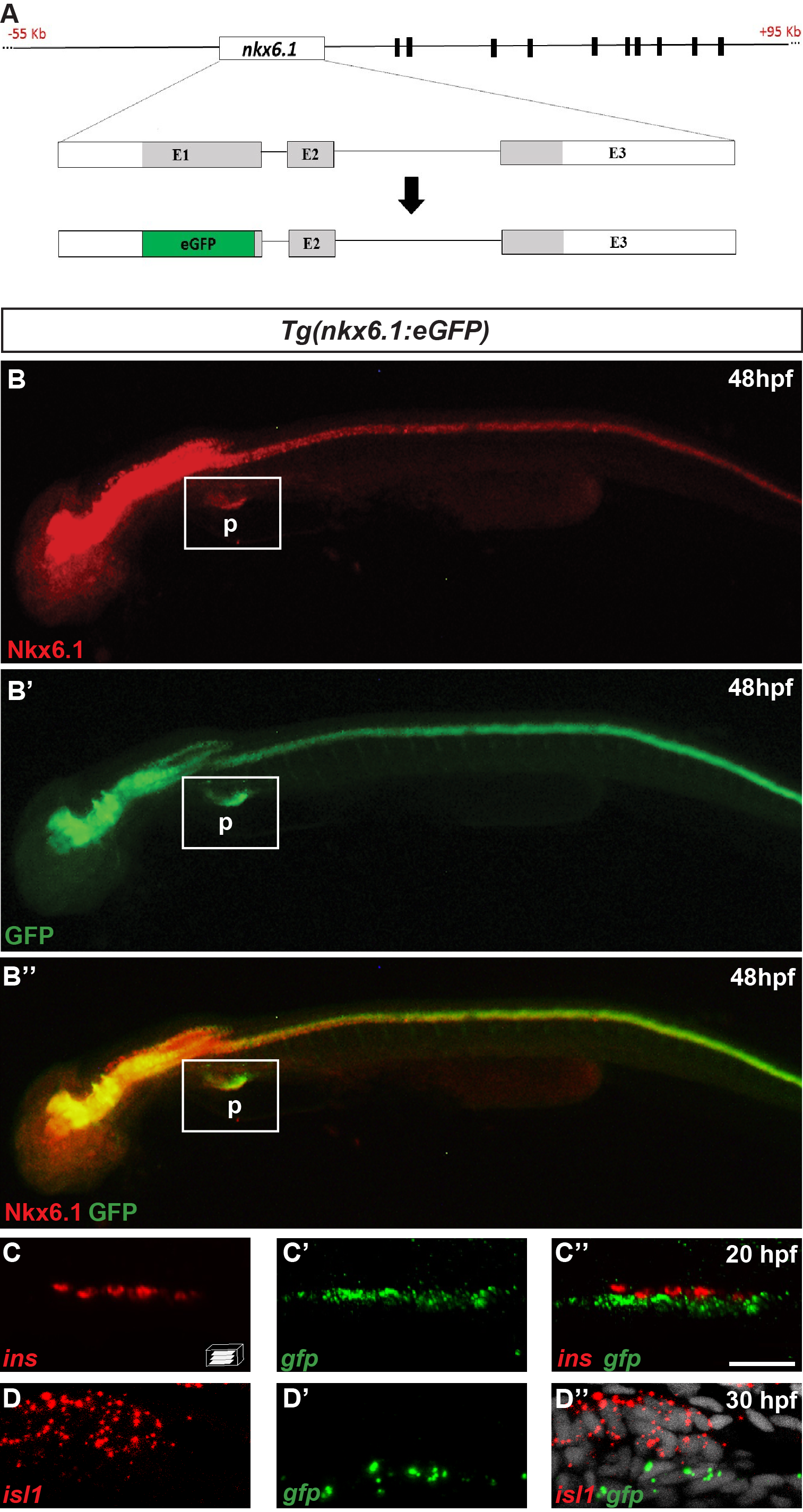

Supplement: Additional file 1: Figure S1. — The bacterial artificial chromosome (BAC) reporter line Tg(nkx6.1:eGFP) mirrors the expression of the endogenous nkx6.1 gene. A Schematic representation of the −55 to +95 kb nkx6.1:eGFP BAC transgene. This BAC includes sequence blocks that are highly conserved amongst vertebrates (black boxes) located from 11 to 77 kb downstream of the nkx6.1 gene. By BAC recombineering using galK selection, the eGFP cassette (green box) was introduced into exon 1, replacing the beginning of the nkx6.1 open reading frame (aa 1 to 149). B Epifluorescence microscopy images of the immunodetection of endogenous Nkx6.1 (red) and GFP (green) in Tg(nkx6.1:eGFP) embryos. Lateral views of 48 hpf embryos with the anterior part to the left. C Confocal projection images of whole mount fluorescent in situ hybridization on a Tg(nkx6.1:eGFP) embryo showing that the gfp transcripts are not expressed in the insulin+ cells at 20 hpf. D Confocal projection images of whole mount fluorescent in situ hybridization on a Tg(nkx6.1:eGFP) embryo showing that the gfp transcripts are not expressed in the isl1+ cells at 30 hpf. Scale bar = 15 μm. P pancreas. (PNG 2609 kb) [file 12915_2015_179_MOESM1_ESM.png]

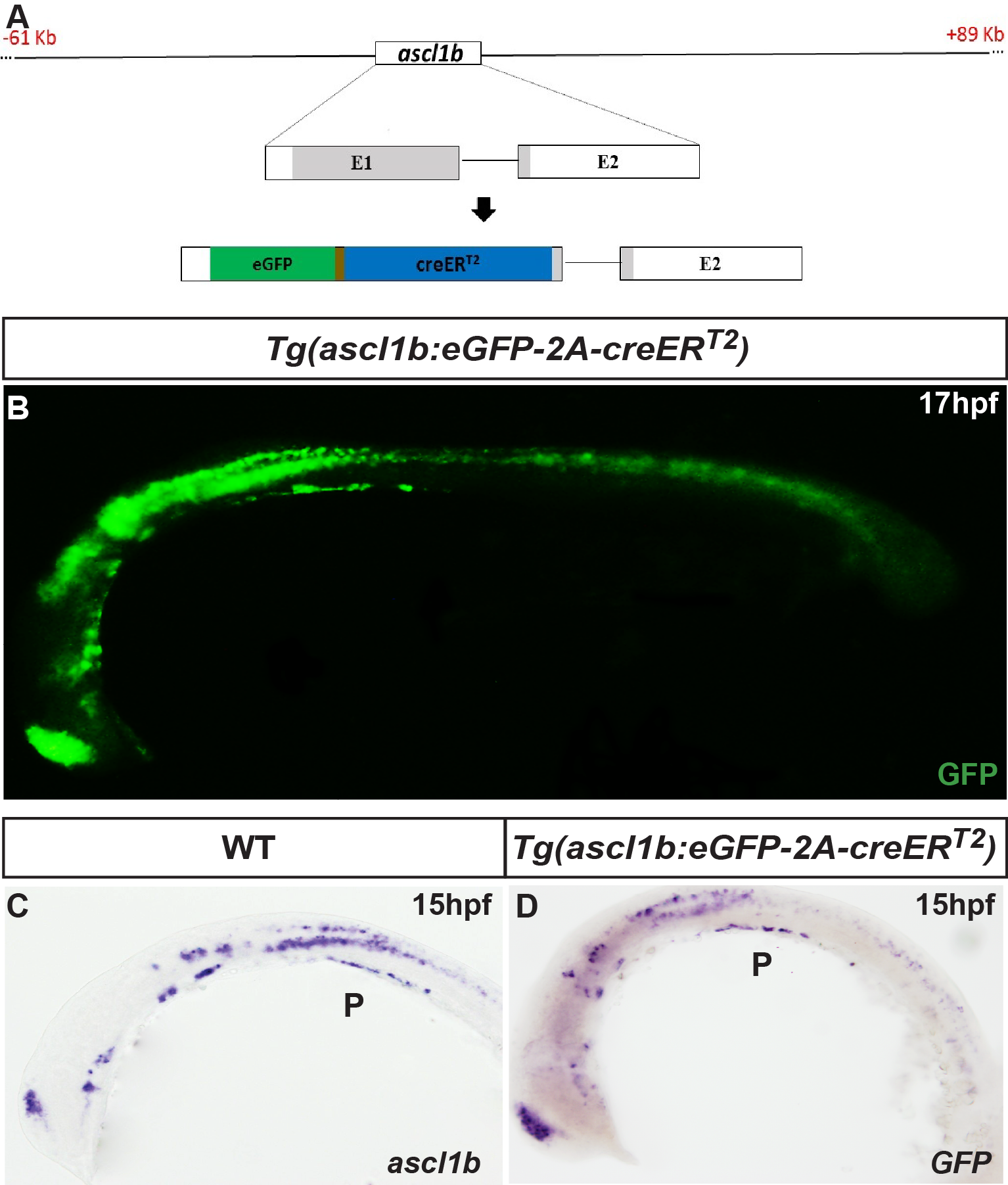

Supplement: Additional file 2: Figure S2. — The bacterial artificial chromosome (BAC) reporter line Tg (ascl1b:eGFP-creER T2 ) mirrors the expression of the endogenous ascl1b gene. A Schematic representation of the −61 to +89 kb ascl1b:eGFP-2A-creER T2 BAC transgene. By BAC recombineering using galK selection, the eGFP-2A-creER T2 cassette is introduced into exon 1, replacing the beginning of the ascl1b open reading frame (aa 1 to 163). B Epifluorescence microscopy images of the immunodetection of GFP in 17-hpf Tg(ascl1b:eGFP-creER T2 ) embryos. C, D Visible WISH showing expression of endogenous ascl1b in a wild-type (WT) embryo (C) and of GFP in Tg(ascl1b:eGFP-creER T2 ) embryos (D) at 15 hpf. Lateral views with the anterior part to the left. P pancreas. (PNG 1068 kb) [file 12915_2015_179_MOESM2_ESM.png]

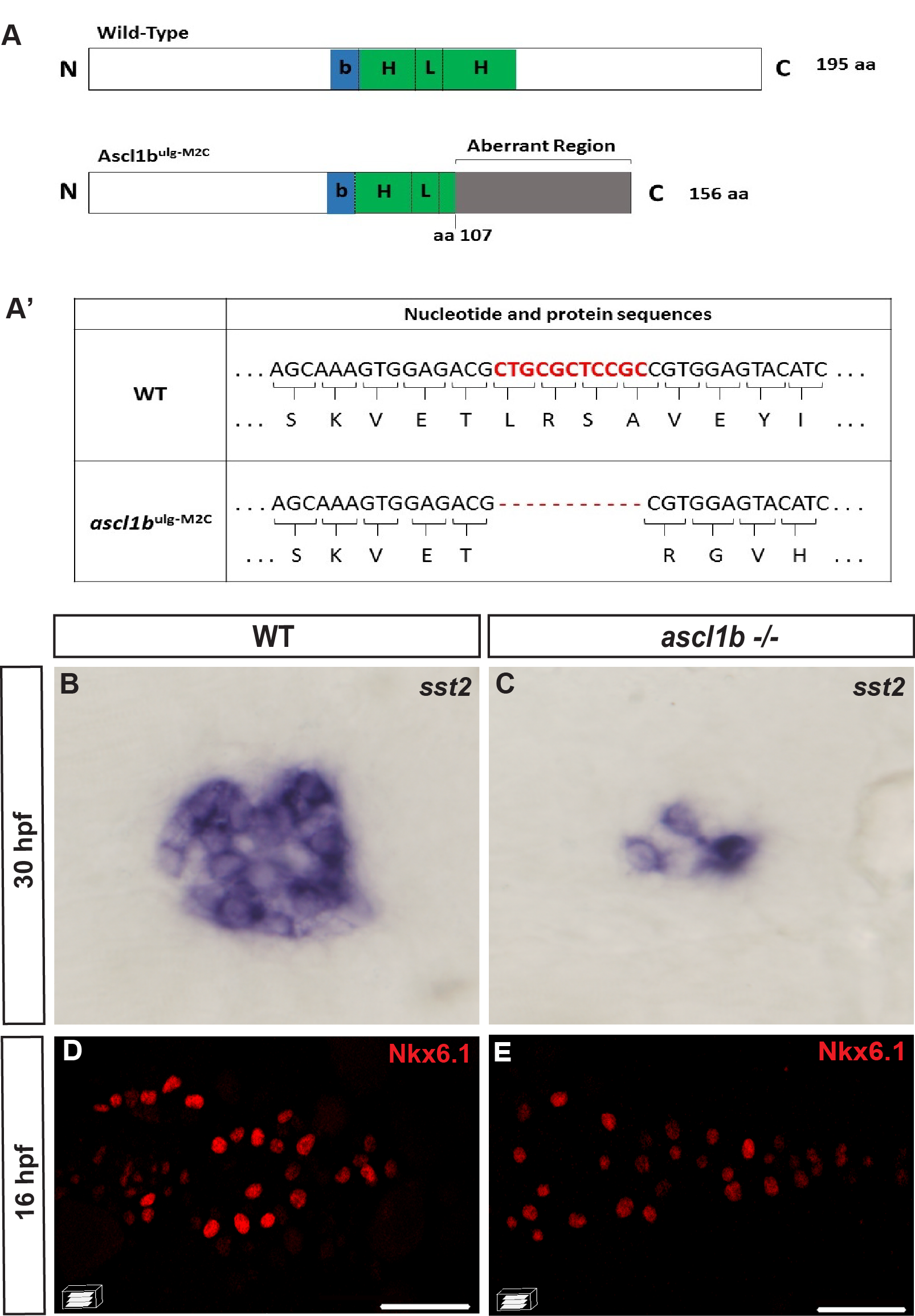

Supplement: Additional file 3: Figure S3. — nkx6.1 expression is not repressed by Ascl1b. A Schematic representation of wild-type (WT) and mutant Ascl1bulg-M2C proteins. The basic domain (b) (+70/+78) is represented by a blue box and the helix loop helix (HLH) domain (+79/+123) by a green box. The coding region of the mutant Ascl1bulg-M2C protein contains an 11-bp deletion after the aa 107 codon, leading to a frameshift and the production of an aberrant region of 48 aa instead of the second helix, known to be essential for the function of the bHLH proteins. A' Table showing part of the nucleotide and protein sequence of the ascl1b gene and of the mutated form in the Ascl1bulg-M2C mutant. B, C WISH showing the drastic reduction of somatostatin expression in the ascl1b-/- mutant compared to the WT embryo at 30 hpf. This phenotype is identical to the one of embryos injected with a translation-blocking morpholino, targeting the translation start site of ascl1b mRNA [15, 77, 78], suggesting that the mutant Ascl1bulg-M2C is effectively a null mutant. D, E Confocal projection images of Nkx6.1 immunodetection showing equivalent number of nkx6.1+ cells in WT and ascl1b -/- mutants at 16 hpf. All views are ventral with the anterior part to the left. Scale bars = 40 μm. (PNG 1321 kb) [file 12915_2015_179_MOESM3_ESM.png]

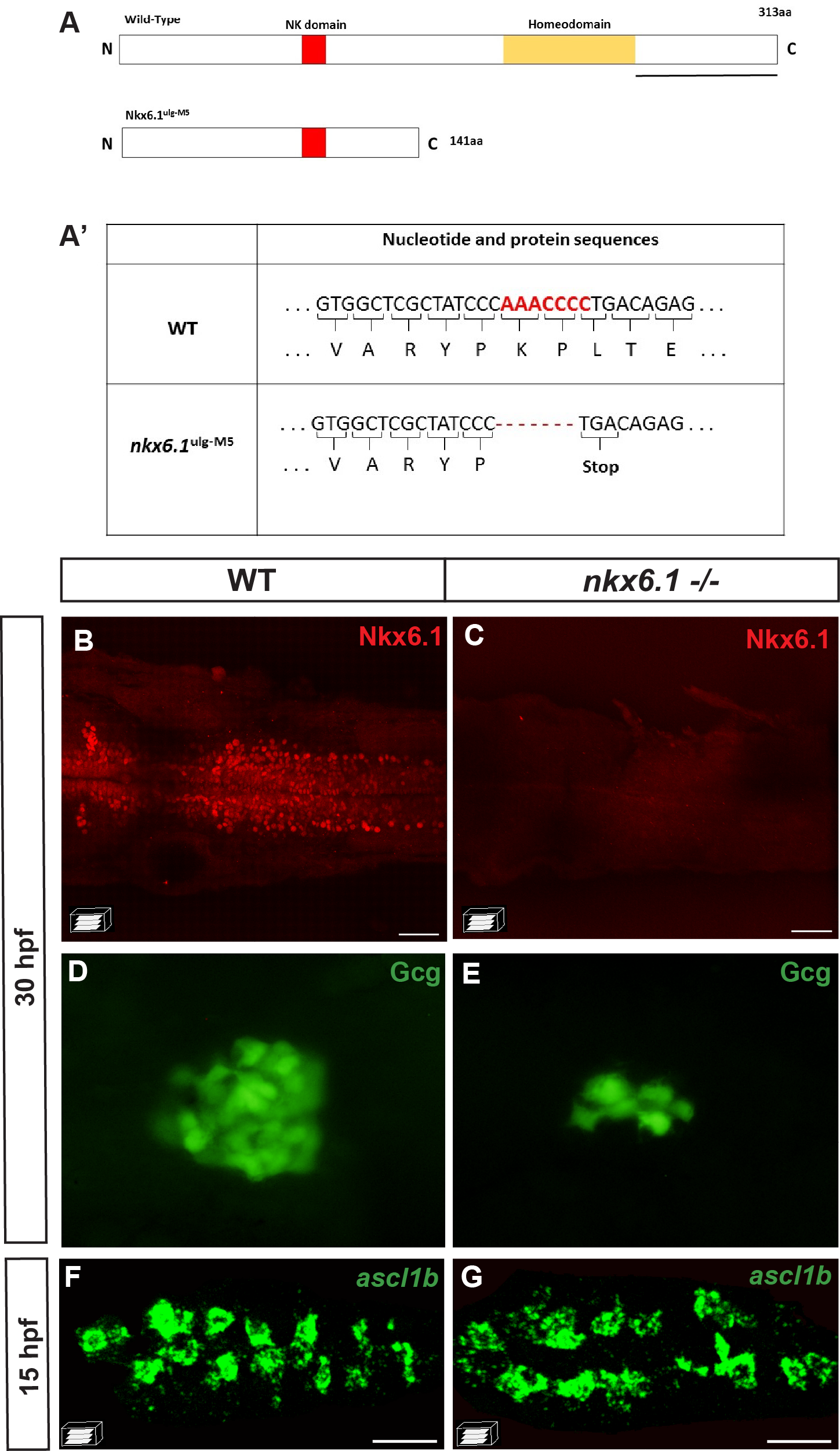

Supplement: Additional file 4: Figure S4. — ascl1b expression is not repressed by Nkx6.1. A Schematic representation of wild-type (WT) and mutant Nkx6.1ulg-M5 proteins with the yellow box representing the homeodomain and the red box representing the NK domain. The coding region of the mutant Nkx6.1ulg-M5 protein contains a 7-bp deletion after the aa 141 codon, leading to the apparition of a STOP codon just after the deletion. The black line represents the region of the protein recognized by the Nkx6.1 antibody. A' Table showing part of the nucleotide and protein sequence of the nkx6.1 gene and of the mutated form in the Nkx6.1ulg-M5 mutant. B, C Nkx6.1 immunodetection showing the loss of the full-length Nkx6.1 protein in the nkx6.1 -/- mutant. D, E Glucagon immunodetection showing a drastic reduction of the number of glucagon-expressing cells in the nkx6.1 -/- mutant compared to the WT embryo at 30 hpf. This phenotype is the same as the one of embryos injected with the MO1 translation-blocking morpholino, targeting the translation start site of nkx6.1 mRNA [18], shown to prevent nkx6.1 expression efficiently in the neural tube [72]. This strongly suggests that the mutant Nkx6.1ulg-M5 is a null mutant. F, G Confocal projection images of fluorescent WISH showing equivalent number of ascl1b+ cells in WT and nkx6.1 -/- mutants at 15 hpf. All views are ventral with the anterior part to the left. Scale bars = 40 μM. (PNG 1527 kb) [file 12915_2015_179_MOESM4_ESM.png]

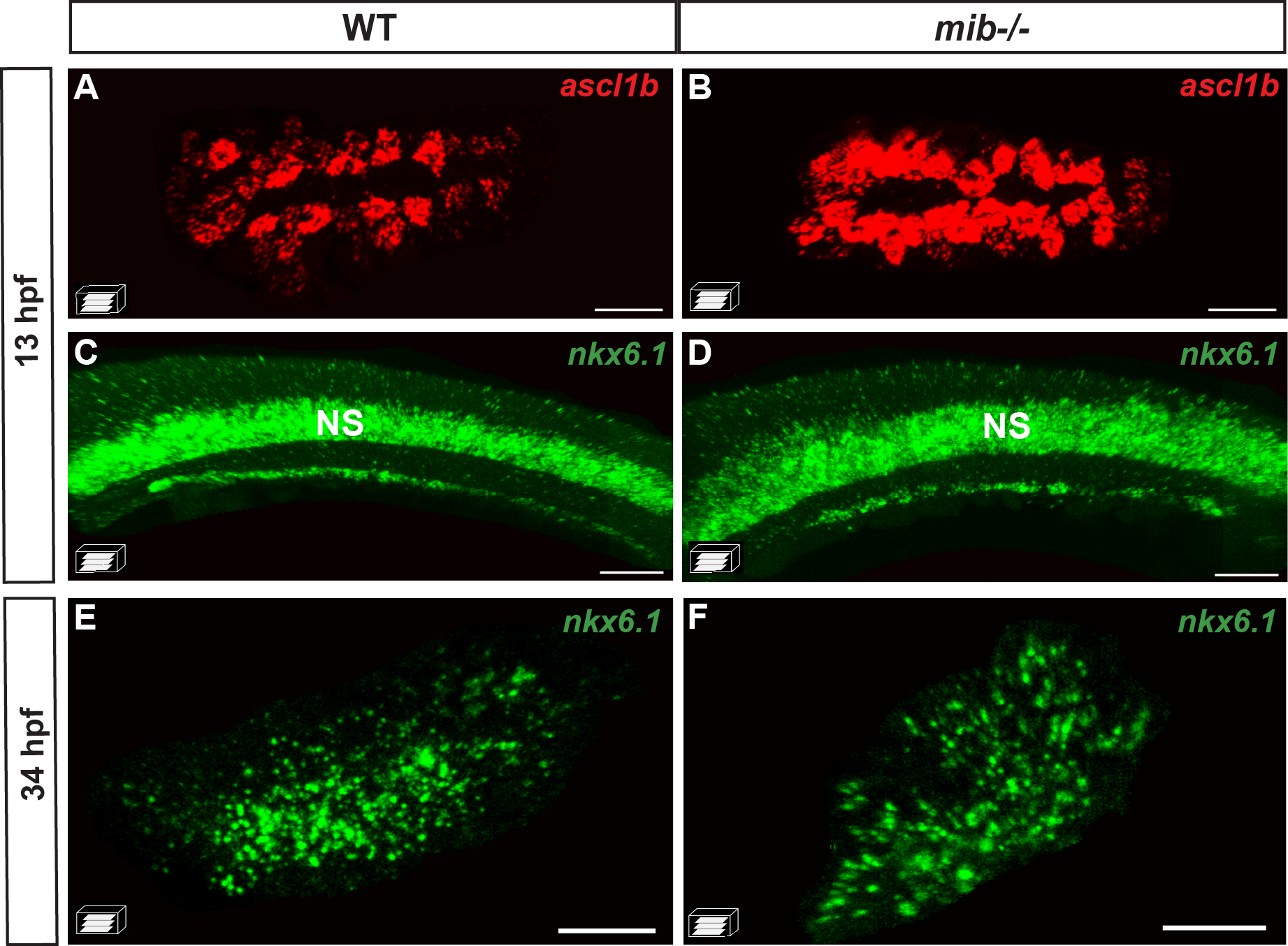

Supplement: Additional file 5: Figure S5. — The initiation of nkx6.1 expression is independent of Notch signaling. A–D Fluorescent WISH showing a drastic increase in the pancreatic dorsal bud of ascl1b expression in the mind bomb (mib -/- ) mutants while nkx6.1 expression is not affected at 13 hpf. E, F Fluorescent WISH showing that the pancreatic expression of nkx6.1 in the ventral bud is not perturbed at 34 hpf in mib -/- mutants. The ventral (A, B, E, F) or lateral (C, D) views represent confocal projection images with the anterior to the left. Scale bars = 40 μM. NS nervous system. (PNG 968 kb) [file 12915_2015_179_MOESM5_ESM.png]

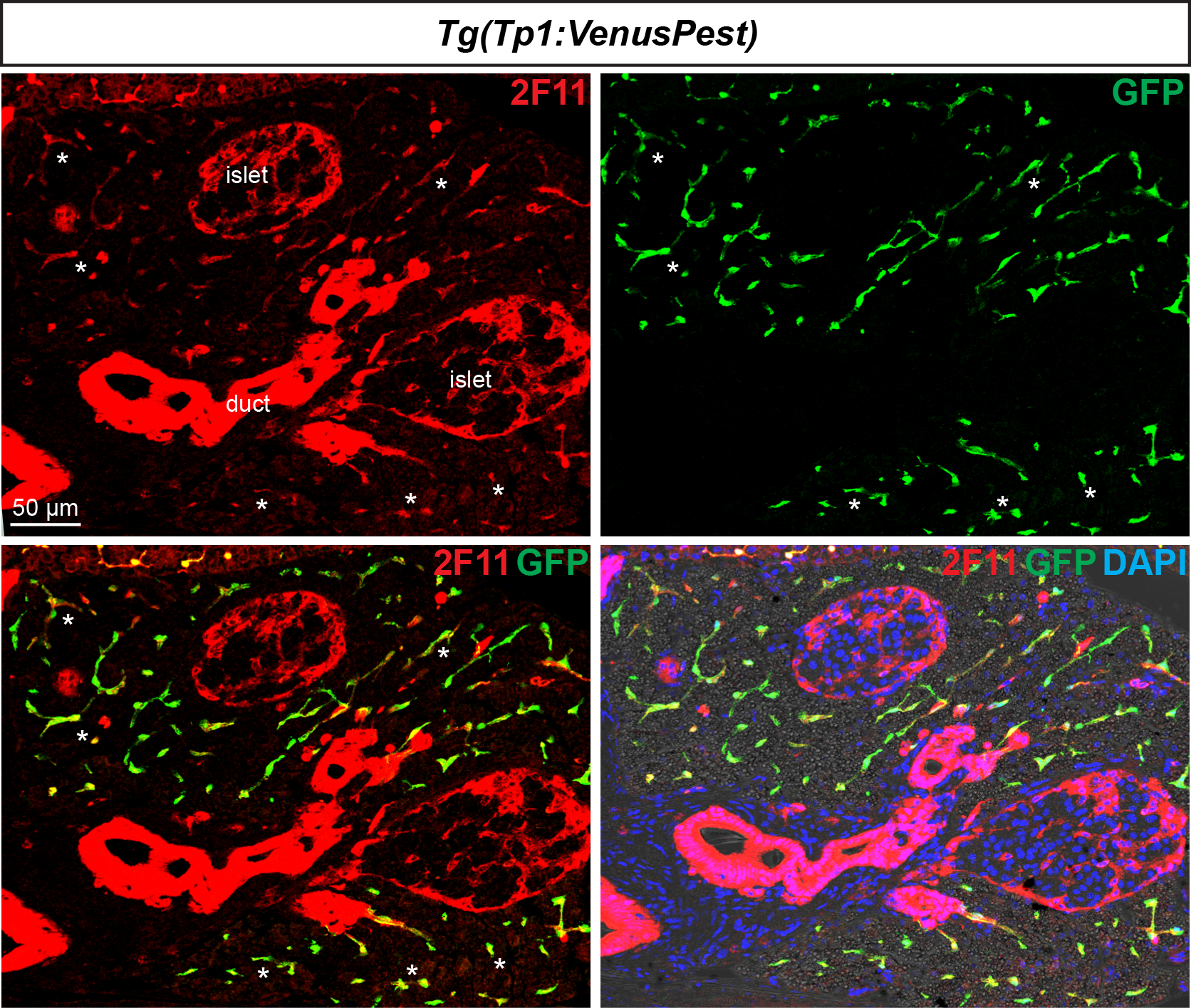

Supplement: Additional file 6: Figure S6. — All Notch-responsive cells in the adult pancreas are CACs. Notch active cells in Tg(Tp1:VenusPest) adult fish were labeled with anti-GFP to visualize VenusPest (green) and the ductal marker 2F11 (red). VenusPest labeling is detected throughout the exocrine tissue (see asterisks highlighting examples) and where it highlights the CACs together with 2F11. In contrast, ductal structures with intense 2F11 staining do not exhibit any Venus+ (Notch on) cells. Note also the presence of 2F11 in endocrine islet cells as previously reported [79]. (PNG 2854 kb) [file 12915_2015_179_MOESM6_ESM.png]

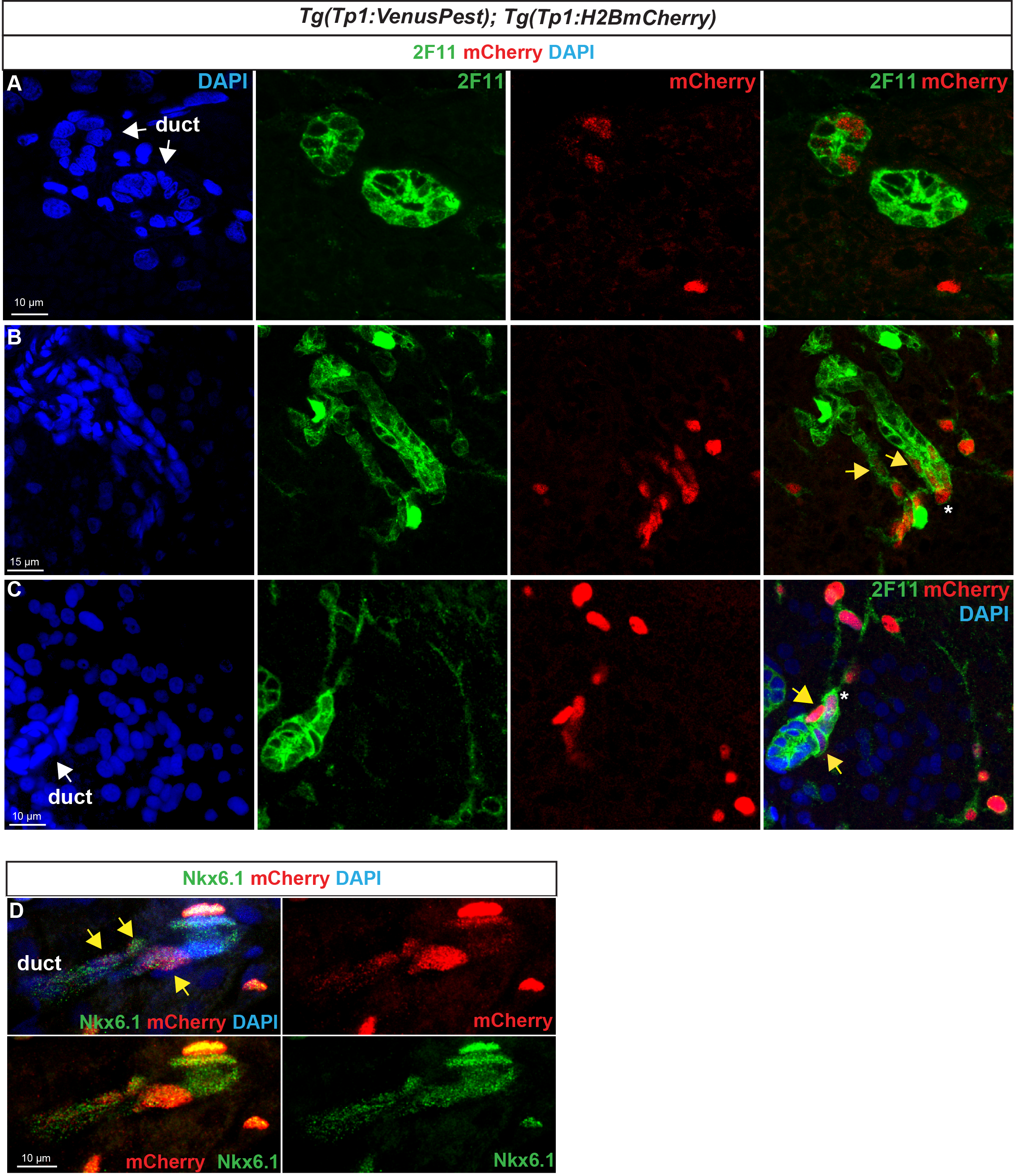

Supplement: Additional file 7: Figure S7. — Notch-responsive terminal end duct cells/CACs give rise to other ductal cells. Immunodetection of H2BmCherry and ductal markers in the pancreas of adult Tg(Tp1:VenusPest); Tg(Tp1:H2BmCherry) zebrafish. A–C H2BmCherry+ cells (red) in ductular structures labeled with the ductal (ducts and CACs) marker 2F11 (green). A, B separate channels of Fig. 8b–c. B, C Weak H2BmCherry labeling is present near the extremity of a terminal (or intercalated) duct (yellow arrows). The asterisks identify a CAC at the tip of the duct (strong H2BmCherry labeling). D Comparison of H2BmCherry (red) with endogenous Nkx6.1 (green) showing ductal Nkx6.1+ cells co-expressing H2BmCherry (yellow arrows). (PNG 4967 kb) [file 12915_2015_179_MOESM7_ESM.png]

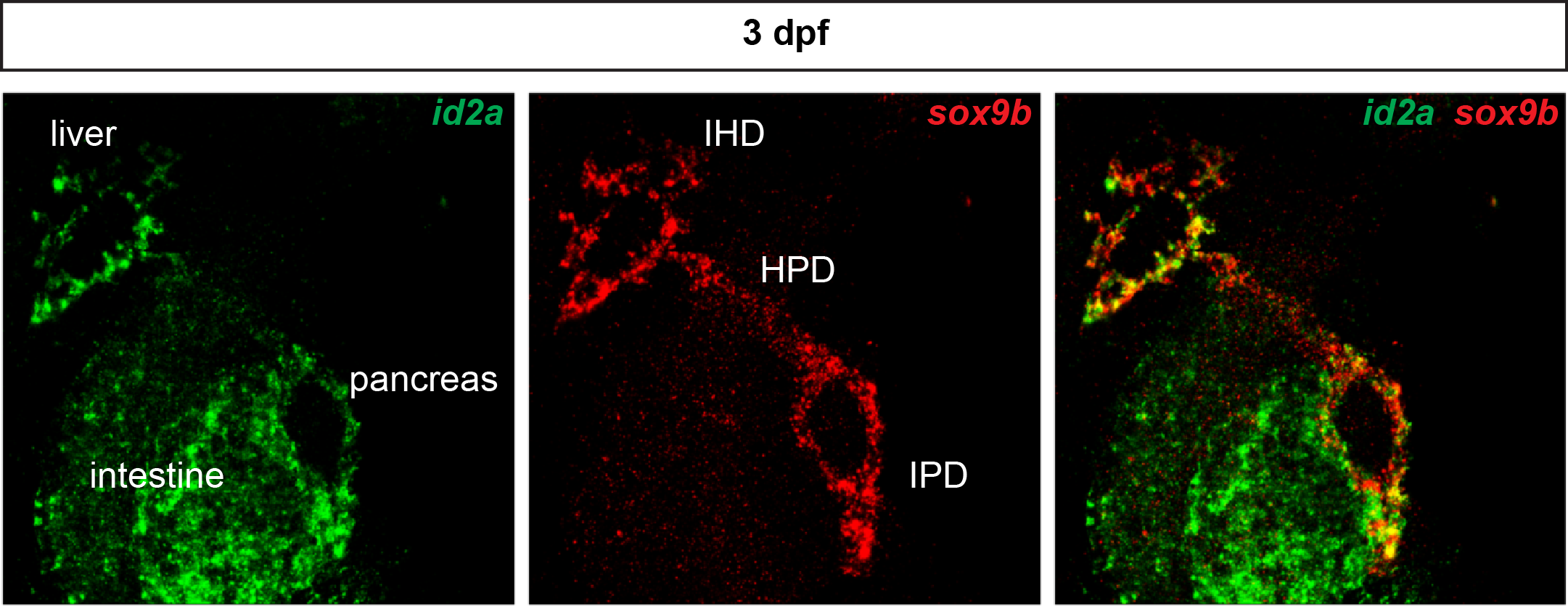

Supplement: Additional file 9: Figure S8. — Ductal expression of id2a during zebrafish development. Whole mount fluorescent in situ hybridization showing id2a (green) and the ductal marker sox9b (red) in a 3-dpf larva. The expression of both genes overlaps as highlighted by yellow labeling. In addition to expression in the intrapancreatic ducts (IPD), id2a is also expressed in the intrahepatic (IHD). HPD hepatopancreatic ducts. (PNG 537 kb) [file 12915_2015_179_MOESM9_ESM.png]
