# Supplementary material for: Progenitor potential of nkx6.1-expressing cells throughout zebrafish life and during beta cell regeneration
Source: BMC Biol. 2015 Sep 2;13:70. doi: 10.1186/s12915-015-0179-4 (PMC4556004; doi:10.1186/s12915-015-0179-4)
Supplement: Additional file 8: Table S1. — Duct-specific genes listed by decreased expression level. We found 293 genes corresponding to the following “specificity” criteria: gene expression level normalized counts >1000 in duct and <1000 in non-ductal (averaged endocrine and acinar) transcriptome and ≥16 fold enrichment in ducts versus non-duct (averaged endocrine and acinar), expressed in log2 ratio (≥4). Ratio is mean of normalized counts in duct transcriptomes/mean of normalized counts in endocrine and in acinar transcriptomes. Known ductal markers and genes involved in the Notch pathway are highlighted in red. Novel markers and genes involved in the Wnt pathway are highlighted in green. (DOCX 26 kb) [file 12915_2015_179_MOESM8_ESM.docx]

| **Gene name** | **log2(ratio D/A+E)** |
| --- | --- |
| ***sb:cb252*** | 8,1 |
| ***phlda2*** | 7,0 |
| ***CR762483.1*** | 7,7 |
| ***tm4sf4*** | 5,9 |
| ***s100a10b*** | 6,2 |
| ***CU596012.2*** | 10,4 |
| ***aqp8a.1*** | 7,3 |
| ***cldn7b*** | 6,7 |
| ***ccl-c5a*** | 6,8 |
| ***s100v2*** | 6,6 |
| ***cd99*** | 6,0 |
| ***si:dkey-238c7.13*** | 7,2 |
| ***cdh17*** | 5,3 |
| ***hmox1a*** | 6,8 |
| ***si:ch211-39i22.1*** | 5,3 |
| ***fam213b*** | 6,8 |
| ***dusp5*** | 7,6 |
| ***pmp22b*** | 5,0 |
| ***dnmbp*** | 4,7 |
| ***hbegfa*** | 6,0 |
| ***EVA1C (7 of 9)*** | 6,5 |
| ***phactr4a*** | 5,2 |
| ***fgl2*** | 5,7 |
| ***sh3pxd2aa*** | 4,4 |
| ***nkx6.1*** | 8,6 |
| ***si:ch211-154o6.6*** | 5,9 |
| ***flna*** | 5,6 |
| ***krt4*** | 5,2 |
| ***frzb*** | 4,8 |
| ***id2a*** | 5,2 |
| ***zgc:113142*** | 9,4 |
| ***slc5a1*** | 7,6 |
| ***ptprjb*** | 6,0 |
| ***stard10*** | 6,6 |
| ***ccl19*** | 7,5 |
| ***gpx1b*** | 6,2 |
| ***ankrd22*** | 7,4 |
| ***zfp36l2*** | 4,8 |
| ***f2rl1.2*** | 5,0 |
| ***rpz5*** | 5,3 |
| ***notch2*** | 4,3 |
| ***tuft1a*** | 5,6 |
| ***ZGC:193706*** | 6,6 |
| ***CABZ01057013.1*** | 5,8 |
| ***nfkbiaa*** | 5,7 |
| ***c5*** | 5,1 |
| ***ZNFX1*** | 6,4 |
| ***ftr82*** | 5,3 |
| ***pttg1ipb*** | 5,6 |
| ***cx30.3*** | 5,1 |
| ***cyp2ad3*** | 5,7 |
| ***nrarpa*** | 6,2 |
| ***cldnh*** | 9,9 |
| ***star*** | 8,3 |
| ***pcnxl3*** | 4,2 |
| ***si:rp71-1h20.9*** | 4,3 |
| ***her6*** | 6,2 |
| ***irf7*** | 5,4 |
| ***RELL1*** | 5,3 |
| ***cfl1l*** | 5,8 |
| ***lima1*** | 5,8 |
| ***CABZ01074367.1*** | 6,0 |
| ***noxo1a*** | 5,4 |
| ***cd74b*** | 4,0 |
| ***si:dkey-79d12.5*** | 4,1 |
| ***itpkcb*** | 6,7 |
| ***si:rp71-1c10.7*** | 5,9 |
| ***timp2b*** | 7,9 |
| ***cmah*** | 4,5 |
| ***ctgfa*** | 5,4 |
| ***her9*** | 4,4 |
| ***si:dkeyp-1h4.9*** | 4,7 |
| ***tinagl1*** | 6,4 |
| ***efhd2*** | 4,4 |
| ***tpm4a*** | 4,8 |
| ***prkcdb*** | 5,1 |
| ***wu:fj08f03*** | 14,6 |
| ***si:rp71-77l1.1*** | 8,8 |
| ***maff*** | 4,9 |
| ***acmsd*** | 4,1 |
| ***klf3*** | 6,2 |
| ***dsc2l*** | 4,0 |
| ***cyr61*** | 4,0 |
| ***muc13b*** | 12,5 |
| ***flnbl*** | 4,3 |
| ***efna1b*** | 5,5 |
| ***oclna*** | 4,1 |
| ***zgc:109868*** | 8,1 |
| ***myh9a*** | 4,3 |
| ***si:ch211-105c13.3*** | 4,2 |
| ***si:ch211-212g7.6*** | 4,2 |
| ***gro2*** | 4,2 |
| ***ptdss1b*** | 6,2 |
| ***pik3cb*** | 4,3 |
| ***frmd4ba*** | 5,3 |
| ***arrdc1a*** | 5,7 |
| ***tnfaip8l3*** | 6,1 |
| ***anxa3b*** | 4,1 |
| ***ccl20*** | 4,1 |
| ***cfhl2*** | 5,2 |
| ***tnfrsf9a*** | 4,5 |
| ***chia.6*** | 4,5 |
| ***ctdsp2*** | 4,6 |
| ***rel*** | 7,9 |
| ***gdpd3a*** | 5,5 |
| ***irf6*** | 4,2 |
| ***ptgis*** | 6,7 |
| ***entpd8*** | 4,7 |
| ***rnasel3*** | 5,8 |
| ***arhgap29a*** | 6,3 |
| ***mpp5a*** | 4,3 |
| ***SYTL2 (1 of 3)*** | 4,2 |
| ***SH3BGRL*** | 4,3 |
| ***mgst1.2*** | 4,1 |
| ***il13ra1*** | 6,1 |
| ***sox11a*** | 5,1 |
| ***rdh10a*** | 4,5 |
| ***si:dkey-29j8.4*** | 8,1 |
| ***tnfrsf1a*** | 4,3 |
| ***slc34a2b*** | 5,7 |
| ***CR774179.2*** | 6,7 |
| ***slc5a8l*** | 10,8 |
| ***lect1*** | 6,3 |
| ***zgc:152791*** | 6,4 |
| ***elovl7a*** | 5,8 |
| ***ctsk*** | 6,5 |
| ***TMC5*** | 5,0 |
| ***enc1*** | 4,7 |
| ***plk2b*** | 5,3 |
| ***wnt7bb*** | 4,0 |
| ***dsg2*** | 4,2 |
| ***tmem45b*** | 4,5 |
| ***sult1st1*** | 7,2 |
| ***si:rp71-17i16.6*** | 4,5 |
| ***metrn*** | 4,3 |
| ***chia.4*** | 7,7 |
| ***epha2a*** | 6,8 |
| ***cfhl4*** | 4,1 |
| ***crb3b*** | 4,2 |
| ***kcnk5b*** | 9,7 |
| ***bin2a*** | 9,1 |
| ***vamp8*** | 4,9 |
| ***CU928070.1*** | 7,2 |
| ***sox9b*** | 8,0 |
| ***cdc25*** | 4,5 |
| ***shroom4*** | 4,4 |
| ***yap1*** | 4,6 |
| ***ldlr*** | 6,5 |
| ***chmp4c*** | 7,9 |
| ***abracl*** | 5,1 |
| ***sfrp5*** | 8,1 |
| ***coch*** | 4,3 |
| ***nrp1a*** | 5,2 |
| ***lats2*** | 5,1 |
| ***crip1*** | 6,5 |
| ***gpa33*** | 5,1 |
| ***si:ch211-269i23.2*** | 5,4 |
| ***pkn2*** | 5,2 |
| ***enc3*** | 4,1 |
| ***enpep*** | 6,1 |
| ***eps8l1*** | 5,7 |
| ***pfkfb3*** | 4,1 |
| ***zgc:158343*** | 4,7 |
| ***PIEZO2 (2 of 5)*** | 5,0 |
| ***NBEAL1*** | 4,0 |
| ***pvrl2l*** | 6,5 |
| ***zgc:92533*** | 8,6 |
| ***COBLL1 (2 of 2)*** | 4,9 |
| ***degs2*** | 4,4 |
| ***btr02*** | 9,0 |
| ***vtnb*** | 4,8 |
| ***LSP1*** | 5,8 |
| ***zgc:153911*** | 7,4 |
| ***ccdc136b*** | 5,5 |
| ***rab13*** | 4,3 |
| ***skap1*** | 7,3 |
| ***adam28*** | 4,3 |
| ***ryr3*** | 4,6 |
| ***nox1*** | 7,4 |
| ***piezo1*** | 4,1 |
| ***sparc*** | 4,5 |
| ***celsr1a*** | 4,4 |
| ***si:dkey-283b1.6*** | 7,8 |
| ***rai14*** | 4,3 |
| ***penkb*** | 5,8 |
| ***zgc:64106*** | 5,9 |
| ***hbegfb*** | 7,2 |
| ***dhrs9*** | 4,3 |
| ***abcc2*** | 7,1 |
| ***FMN1 (4 of 4)*** | 5,5 |
| ***myl9a*** | 9,4 |
| ***CABZ01102038.1*** | 5,8 |
| ***si:rp71-36a1.5*** | 4,7 |
| ***MARVELD3*** | 4,6 |
| ***gna12a*** | 5,4 |
| ***itpk1*** | 6,4 |
| ***si:dkey-19a16.4*** | 5,3 |
| ***LRCH1*** | 4,6 |
| ***rnd3b*** | 6,2 |
| ***SLC46A2*** | 5,8 |
| ***pcdh7b*** | 4,0 |
| ***EPHA2 (2 of 2)*** | 4,4 |
| ***emilin1a*** | 6,0 |
| ***amotl2b*** | 7,4 |
| ***zgc:162183*** | 7,1 |
| ***mid1ip1a*** | 6,7 |
| ***swap70b*** | 4,3 |
| ***ugt5b4*** | 6,1 |
| ***cxcl-c1c*** | 4,9 |
| ***slc6a14*** | 11,2 |
| ***ZC3H12A (2 of 2)*** | 4,0 |
| ***zgc:109934*** | 4,1 |
| ***sult6b1*** | 5,4 |
| ***prr5*** | 4,2 |
| ***sh2d5*** | 4,8 |
| ***bckdhbl*** | 5,1 |
| ***boc*** | 5,2 |
| ***slc38a5a*** | 7,4 |
| ***si:dkey-66i24.9*** | 6,5 |
| ***fhl1b*** | 4,3 |
| ***SHROOM3*** | 4,6 |
| ***rbms1a*** | 4,1 |
| ***tac4*** | 9,3 |
| ***FMN1 (3 of 4)*** | 6,7 |
| ***serpinh1a*** | 7,3 |
| ***TNFRSF14 (3 of 12)*** | 5,1 |
| ***slc23a1*** | 5,6 |
| ***il15*** | 4,2 |
| ***btk*** | 5,1 |
| ***cxcr3.1*** | 5,1 |
| ***SOWAHD*** | 4,9 |
| ***fn1a*** | 4,7 |
| ***sult1st3*** | 8,3 |
| ***hsd20b2*** | 4,6 |
| ***pard6a*** | 4,6 |
| ***tmem238*** | 5,1 |
| ***grap2a*** | 4,0 |
| ***si:ch211-178n15.1*** | 4,1 |
| ***CLDN4 (3 of 7)*** | 6,7 |
| ***igfbp5b*** | 4,6 |
| ***thbs1b*** | 6,2 |
| ***CT583700.1*** | 5,6 |
| ***her15.1*** | 4,0 |
| ***EHBP1L1 (1 of 2)*** | 7,2 |
| ***zgc:64022*** | 7,6 |
| ***capn5a*** | 4,1 |
| ***tmem72*** | 5,8 |
| ***zgc:113314*** | 4,6 |
| ***si:ch1073-185p12.2*** | 4,8 |
| ***fam60a*** | 4,0 |
| ***ppl*** | 4,1 |
| ***rab32a*** | 4,2 |
| ***ST14 (2 of 3)*** | 5,6 |
| ***ccdc80*** | 5,6 |
| ***eps8l3*** | 5,4 |
| ***txn*** | 5,5 |
| ***tbata*** | 5,9 |
| ***ugt1b5*** | 6,4 |
| ***ildr1a*** | 7,1 |
| ***itgb4*** | 5,8 |
| ***plekhg2*** | 4,3 |
| ***acp5a*** | 7,9 |
| ***tcf7l1b*** | 5,9 |
| ***tspan13a*** | 4,3 |
| ***cd151l*** | 4,4 |
| ***si:ch1073-228p2.2*** | 5,2 |
| ***ITGA2 (2 of 2)*** | 5,0 |
| ***asz1*** | 6,4 |
| ***marveld3*** | 4,3 |
| ***plekhf1*** | 9,7 |
| ***onecut1*** | 4,8 |
| ***ros1*** | 9,3 |
| ***erbb3a*** | 5,0 |
| ***fa2h*** | 4,6 |
| ***btr12*** | 9,0 |
| ***psmb10*** | 5,3 |
| ***emp2*** | 8,0 |
| ***cldnk*** | 14,5 |
| ***CR339041.1*** | 5,6 |
| ***CDKN1C (2 of 2)*** | 5,4 |
| ***CDH26*** | 4,0 |
| ***ackr4b*** | 4,9 |
| ***fxyd6l*** | 8,4 |
| ***sh3yl1*** | 4,5 |
| ***BX005395.1*** | 6,3 |
| ***XAF1*** | 5,1 |
| ***zgc:110410*** | 8,1 |
| ***anxa5b*** | 4,3 |
| ***CCDC64B*** | 7,4 |
| ***flrt2*** | 6,3 |
| ***igf2bp3*** | 7,0 |
| ***vstm2l*** | 4,7 |
| ***elovl2*** | 4,5 |
