# Supplementary material for: Progenitor potential of nkx6.1-expressing cells throughout zebrafish life and during beta cell regeneration
Source: BMC Biol. 2015 Sep 2;13:70. doi: 10.1186/s12915-015-0179-4 (PMC4556004; doi:10.1186/s12915-015-0179-4)
Supplement: Additional file 10: Table S2. — List of the primers used in this study. The 50 nucleotides added to allow homologous recombination are indicated in capital letters. (DOCX 12 kb) [file 12915_2015_179_MOESM10_ESM.docx]

| List of the primers | |
| --- | --- |
| **O180F** | 5'-GTCCTGCTTCTTGCGCGATATTTAGTTAAAGTTGTAGTTTGTGTCTTGGTTcctgttgacaattaatcatcggca-3' |
| **O253R** | 5'-**G**CATCTCTCCAGTGTGGGCTTTGCATAACTCCAGGCCAGAATATCGGGGTTtcagcactgtcctgctcctt-3’ |
| **O186F** | 5’-GTCCTGCTTCTTGCGCGATATTTAGTTAAAGTTGTAGTTTGTGTC**TTG**GTTatggtgagcaagggcgaggag-3’ |
| **O256R** | 5’-GCATCTCTCCAGTGTGGGCTTTGCATAACTCCAGGCCAGAATATCGGGGTTttacttgtacagctcgtccatg-3’ |
| **O275F** | 5’-GCGCATTACTCGCAAACTCCCCCTCTATTTATCTATTTATTTCACGCGACcctgttgacaattaatcatcggca-3’ |
| **O276R** | 5’-CGAACCATGTGGTGAAGTCCAGCAGTTCTTGCTCCTCGGAGCTGAGATGCtcagcactgtcctgctcctt-3’ |
| **O277F** | 5’-GCGCATTACTCGCAAACTCCCCCTCTATTTATCTATTTATTTCACGCGACatggtgagcaagggcgaggag-3’ |
| **O278R** | 5’-CGAACCATGTGGTGAAGTCCAGCAGTTCTTGCTCCTCGGAGCTGAGATGCtcaagctgtggcagggaaaccc-3’ |
| **O215F** | 5’-ttctctgtttttgtccgtggaatgaacaatggaagtccgagctcatcgctccctgctcgagccgggcccaagtg-3’ |
| **O216R** | 5’-agccccgacacccgccaacacccgctgacgcgaaccccttgcggccgcatattatgatcctctagatcagatc-3’ |
